# *GREM1* is associated with metastasis and predicts poor prognosis in ER-negative breast cancer patients

**DOI:** 10.1186/s12964-019-0467-7

**Published:** 2019-11-06

**Authors:** Ulrike Neckmann, Camilla Wolowczyk, Martina Hall, Eivind Almaas, Jiang Ren, Sen Zhao, Bjarne Johannessen, Rolf I. Skotheim, Geir Bjørkøy, Peter ten Dijke, Toril Holien

**Affiliations:** 1 Centre of Molecular Inflammation Research, Department of Clinical and Molecular Medicine, Faculty of Medicine and Health Sciences, NTNU - Norwegian University of Science and Technology, Trondheim, Norway; 2 Department of Biomedical Laboratory Science, Faculty of Natural Sciences, NTNU - Norwegian University of Science and Technology, Trondheim, Norway; 3 Clinic of Laboratory Medicine, St Olavs Hospital, Trondheim, Norway; 4 Department of Biotechnology and Food Science, Faculty of Natural Sciences, NTNU - Norwegian University of Science and Technology, Trondheim, Norway; 5 K.G. Jebsen Center for Genetic Epidemiology, Department of Public Health and General Practice, Faculty of Medicine and Health Sciences, NTNU - Norwegian University of Science and Technology, Trondheim, Norway; 6 Department of Cell and Chemical Biology, Oncode Institute, Leiden University Medical Center, Leiden, The Netherlands; 7 Department of Molecular Oncology, Institute for Cancer Research, Oslo University Hospital-Radiumhospitalet, Oslo, Norway; 8 Department of Informatics, University of Oslo, Oslo, Norway; 9 Department of Clinical and Molecular Medicine (IKOM), NTNU, Gastro Center, Prinsesse Kristinas gt 1, 7030 Trondheim, Norway; 10 Department of Hematology, St. Olavs Hospital, Trondheim, Norway

**Keywords:** 4T1 model, 66cl4, 67NR, BMP4, GREM1, Metastasis, Gremlin 1

## Abstract

**Background:**

In breast cancer, activation of bone morphogenetic protein (BMP) signaling and elevated levels of BMP-antagonists have been linked to tumor progression and metastasis. However, the simultaneous upregulation of BMPs and their antagonist, and the fact that both promote tumor aggressiveness seems contradictory and is not fully understood.

**Methods:**

We analyzed the transcriptomes of the metastatic 66cl4 and the non-metastatic 67NR cell lines of the 4T1 mouse mammary tumor model to search for factors that promote metastasis. CRISPR/Cas9 gene editing was used for mechanistic studies in the same cell lines. Furthermore, we analyzed gene expression patterns in human breast cancer biopsies obtained from public datasets to evaluate co-expression and possible relations to clinical outcome.

**Results:**

We found that mRNA levels of the BMP-antagonist *Grem1*, encoding gremlin1, and the ligand *Bmp4* were both significantly upregulated in cells and primary tumors of 66cl4 compared to 67NR. Depletion of gremlin1 in 66cl4 could impair metastasis to the lungs in this model. Furthermore, we found that expression of *Grem1* correlated with upregulation of several stem cell markers in 66cl4 cells compared to 67NR cells. Both in the mouse model and in patients, expression of *GREM1* associated with extracellular matrix organization, and formation, biosynthesis and modification of collagen. Importantly, high expression of *GREM1* predicted poor prognosis in estrogen receptor negative breast cancer patients. Analyses of large patient cohorts revealed that amplification of genes encoding BMP-antagonists and elevation of the corresponding transcripts is evident in biopsies from more than half of the patients and much more frequent for the secreted BMP-antagonists than the intracellular inhibitors of SMAD signaling.

**Conclusion:**

In conclusion, our results show that *GREM1* is associated with metastasis and predicts poor prognosis in ER-negative breast cancer patients. Gremlin1 could represent a novel target for therapy.

## Background

Members of the transforming growth factor β (TGF-β) family, including the TGF-βs, activins, nodal, bone morphogenetic proteins (BMPs), and growth and differentiation factors (GDFs), play important roles in tumor progression and formation of metastases [1]. However, these signaling molecules have pleiotropic effects in cancer, as seen with TGF-β signaling, which has been linked to both tumor suppression and tumor promotion depending on the tumor stage [2, 3]. After binding of the TGF-βs to transmembrane receptors, SMAD2 and SMAD3 become phosphorylated and bind to SMAD4; subsequent translocation of this complex to the nucleus result in the expression of TGF-β controlled genes. In contrast, BMP signaling primarily occurs via SMAD1/5/8 instead of SMAD2/3. Receptor activation by TGF-β family ligands can also result in induction of non-SMAD pathways, including mitogen-activated protein kinases (MAPK), and PI3K-Akt-mTOR pathways [4]. Like TGF-βs, BMPs have been implicated in both tumor suppression and tumor progression [5]. Understanding the role of TGF-β family members in cancer and the development of TGF-β targeted therapies have been hampered by the complex interplay of these signaling molecules with intracellular SMAD-inhibitors and extracellular BMP-antagonists. The expression of intracellular inhibitory SMADs, i.e. SMAD6 and SMAD7, is induced in response to several TGF-β ligands and form a negative feedback loop. Extracellular BMP-antagonists, like noggin, gremlin1, Dand5, and follistatin, bind to BMPs and prevent binding of the BMPs to their respective receptor [6, 7]. Like BMPs and TGF-βs, also BMP-antagonists can have a tumor-promoting role. For instance, Dand5, also known as Coco, facilitated formation of lung metastases in the 4T1 mammary tumor model by blocking lung-derived BMPs [8], whereas gremlin1 promoted stem cell maintenance in both glioma [9] and colorectal cancer [10].

Metastasis is a complex multi-step process, including local invasion, intravasation of tumor cells into the circulation, tumor cell dissemination, extravasation at metastatic site, survival at secondary site, and finally proliferation and formation of metastases [11]. To metastasize, cancer cells of epithelial origin have to acquire new properties such as increased motility, anchorage-independent growth and the ability to adapt to a foreign microenvironment. Only cancer cells that display a high degree of phenotypic plasticity and thus are able to adapt to stressful conditions and changing environments, will survive. For example, migratory tumor cells require stem cell-like properties including a low differentiation status [12].

The immunocompetent mouse model for metastatic mammary carcinoma called 4T1 consists of several cell lines isolated from the same spontaneously formed tumor [13, 14]. All the cell lines form primary tumors when injected into the fat pad of BALB/c mice, but they differ widely in their ability to form distant metastasis. Here, we focused on 67NR, which does not metastasize, and 66cl4, which metastasizes to the lungs. We hypothesized that their different metastatic propensity relates to differences in the secretome of the two cell lines, since cancer cell-derived factors influence the phenotype of the cancer cells and/or the stroma cells. We therefore performed transcriptome analysis of 66cl4 and 67NR cells from in vitro culture and grown as tumors in BALB/c nude mice and focused on differentially expressed mRNAs encoding secreted proteins. Expression of the BMP-antagonist *Grem1* was significantly upregulated in 66cl4 compared to 67NR both in cell culture and primary tumors. Gremlin1 binds and inhibits BMP2, BMP4, and BMP7 [15], which play important roles during cellular differentiation [1]. In line with the elevated expression of *Grem1* in 66cl4, several stem cell markers were upregulated in the 66cl4 cells. Interestingly, compared to the non-metastatic 67NR cells, the metastatic 66cl4 cells also expressed significantly elevated levels of *Bmp4*. Consistent with a role of these secreted proteins in metastasis, we found that high *GREM1* expression correlated with reduced relapse-free survival (RFS) in estrogen receptor (ER)-negative breast cancer patients and that the association with poor prognosis remains, even with elevated co-expression of a BMP in the tumor.

## Methods

For information about Transcriptome analysis; Quantitative real-time PCR; Immunoblotting; Harvest of conditioned medium; ELISA; In vitro extravasation assay; Cell proliferation assay; Soft-agar assay; Flow cytometry; see Additional file 1: Supplementary Methods.

### Cell culture and generation of stable cell lines

The mouse mammary tumor cell lines 67NR and 66cl4 cell lines were obtained from Barbara Ann Karmanos Cancer Institute, Detroit, MI, USA. The cells were cultured in DMEM (Lonza, BioWhittaker, BE12- 604F) supplemented with 10% fetal calf serum (FCS, Thermo Fischer Scientific, #10270–106), 2 mM L-Glutamine (Lonza Group, De-17-605E) and 50 U/ml penicillin-streptomycin (ThermoFischer Scientific, Gibco, #15070–063). Primary umbilical vein endothelial cells, normal, human (HUVEC) were obtained from ATCC (ATCC-PCS-100-010). The cells were cultured in Medium 200 (Gibco, M200500) supplemented with low serum growth supplement (LSGS) (Gibco, S00310). All cells were incubated at 37 °C with 5% CO_2_.

CRISPR/Cas9-*GREM1* knockouts and CRISPR/Cas9 vector control cell lines were generated by viral transduction. The mouse lentiviral particles were purchased from Sigma Aldrich (CRISPR/Cas9-GREM1 Mm0000325632 and Mm0000325634; CRISPR/Cas9-NT, CRISPR12V-1EA). 6 h after seeding, 66cl4 cells were infected with lentiviral particles (MOI 0.1) in medium containing hexadimethrine bromide (8 μg/ml). After 24 h cells were split 1:17. Starting 48 h after infection, cells were selected with puromycin (3.25 μg/ml) for 1 week. The selection medium was replaced every 2–3 days. Single cell colonies were picked using cloning cylinders and tested for gremlin1 expression levels by western blot. The CRISPR/Cas9 NT_mix was generated by trypsinizing a plate containing thousands of puromycin resistant colonies.

### Orthotopic mouse tumors and in vivo lung colonization assay

For all experiments, female mice (8–11 weeks old, Janvier Labs, France) were used. For orthotopic tumors, mice were anaesthetized and injected with 5 × 10^5^ viable cells resuspended in PBS into the fourth mammary fat pad. When palpable, tumor size was measured twice weekly using electronic calipers. Tumor volume was calculated: V_T_ = (length x width^2^) / 2. NT controls and gremlin1 depleted cell lines were injected into nude mice (BALB/cAnNRj-*Foxn1*^*nu/nu*^), *n* = 10 per cell line. Mice were sacrificed after 21 days, unless otherwise stated. Weight of primary tumors and lungs was recorded. For the in vivo lung colonization assay female mice were anaesthetized and injected with 5 × 10^5^ cells/100 μl PBS in the lateral tail vein. Mice were monitored daily and sacrificed 15 days after injection. Entire lungs were weighed.

### Zebrafish xenograft model

The transgenic fish line Tg(*fli1:GFP*) was used [16, 17]. The tumor cell lines were fluorescently labelled with mCherry using plv-mCherry lentiviruses. G418 was used as selection marker. Embryo preparation and tumor cell implantation was done as previously described [18]. Briefly, Tg(*fli1:GFP*) zebrafish embryos were dechorionated 2 days post fertilization. Single cell suspensions of 66cl4 wildtype cells and variants were re-suspended in PBS and kept at 4 °C until transplantation. Cell suspensions were loaded into borosilicate glass capillary needles (1 mm O.D. × 0.78 mm I.D.; Harvard Apparatus) and injected into the duct of Cuvier (DoC) using a Pneumatic Picopump and a manipulator (WPI, Stevenage, UK). After injection of approximately 400 cells, zebrafish embryos were maintained at 33 °C. Invasive clusters were quantified 6 days post-implantation (dpi). Zebrafish embryos were fixated with 4% paraformaldehyde at 4 °C overnight and were imaged in PBS using a Leica SP5 STED confocal microscope. Confocal stacks were processed for maximum intensity projections with Image J. Brightness and contrast of images were adjusted with Adobe Photoshop CS6. For each cell line, at least fifty embryos were analyzed.

### Gene co-expression network analysis

RNA-seq data were downloaded from the study by Ciriello et al. in Cell, 2015 [19] from The Cancer Genome Atlas (TCGA; http://cancergenome.nih.gov). The cohort consists of 817 cases of breast cancer with 17,214 genes, normalized within samples to the median gene expression. Due to missing values, only 421 cases and 16,749 genes were available for download and analysis. To assess the association between *GREM1* and other genes, an ego-centric network with *GREM1* in the center was created using sample Pearson correlation. The sample Pearson correlation represents the similarity between *GREM1* and the corresponding gene, in terms of linear correlation. The correlations range from − 1 to 1, where a value of 1 represents a total positive linear correlation, e.g. similar expression values of *GREM1* and the corresponding gene, − 1 represents a total negative linear correlation, e.g. opposite expression values of *GREM1* and the corresponding gene. A value near zero represents no linear correlation, e.g. no linear association between the expression values of *GREM1* and the corresponding gene.

### KM plotter

KM plotter (http://kmplot.com/analysis/) is an online tool for examining prognostic markers in breast cancer subtypes, which utilizes data from multiple cDNA microarray experiments [20, 21]. Survival analysis (RFS or OS) was done using the KM plotter database 2017 version. High and low expression were defined as above (hazard ratio (HR) > 1.2, *p*-value < 0.05) and below (HR < 0.83, p-value < 0.05) median, except for the OS analysis where best cutoff was used.

### cBioPortal

cBioPortal (http://www.cbioportal.org/) is an open-access database that allows visualization and analysis of large-scale cancer genomics data sets [22, 23]. Our analyses utilize the OncoPrints visualization to identify genomic alterations, including somatic mutations, mRNA expression and amplifications across a set of cases. This visualization shows the genes as rows, while individual cases are shown as columns. For this analysis we used the TCGA Provisional data set for invasive breast carcinoma, and selected mutations, putative copy-number alterations and mRNA expression as genomic profiles.

### Gene enrichment analysis

Gene enrichment analyses were done with the online tools Enrichr (http://amp.pharm.mssm.edu/Enrichr/) [24, 25] for the 66cl4/67NR transcriptome data (Fig. 2b) and Reactome (https://reactome.org/) [26] for the TCGA cohort (Fig. 3b).

### Co-expression analysis

Gene co-expression analysis was performed using the online gene co-expression search engine SEEK (Search-Based Exploration of Expression Compendium [Human]) (http://seek.princeton.edu/) [27] searching 331 breast cancer patient data sets. In addition, both Expression Atlas (https://www.ebi.ac.uk/gxa) [28] and CCLE (Broad Institute Cancer Cell Line Encyclopedia, https://portals.broadinstitute.org/ccle) [29] was used for gene expression analysis in human breast cancer cell lines.

### Statistics

Statistical analyses were performed in GraphPad Prism 7. Values are expressed as mean ± standard deviation (SD), or mean ± standard error of the mean (SEM). Statistical analyses were performed by paired 2-tailed Student’s t-test after log-transformation (*0.01 < *P* < 0.05, **0.001 < *P* < 0.01, *** *P* < 0.001).

## Results

### *Grem1* is highly expressed in the metastatic 66cl4 cells and elevated *GREM1* in tumor biopsies correlates with reduced relapse-free survival in patients

**Fig. 1:**
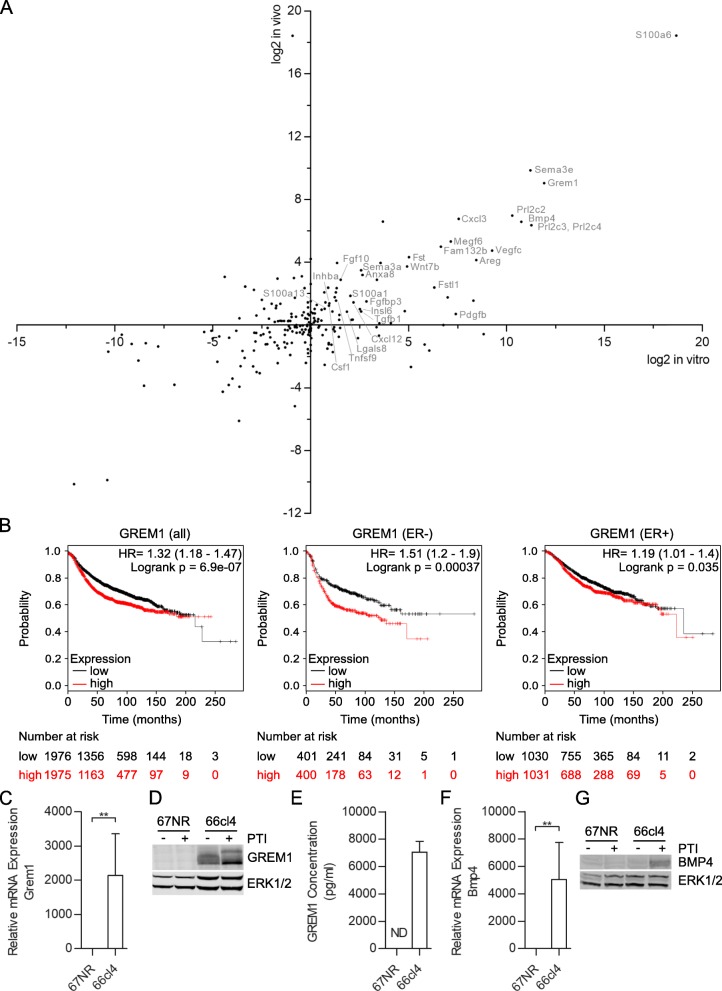
*Grem1* is highly expressed in 66cl4 and *GREM1* correlates with RFS in breast cancer patients. **(a)** Scatter plot of in vivo versus in vitro differential expression of mRNAs encoding secreted proteins in 66cl4 and 67NR. A positive number indicates higher expression in 66cl4, whereas a negative number indicates higher expression in 67NR. The 28 factors were significantly overexpressed in both 66cl4 cells and 66cl4 primary tumors (cut-off: fold-change ≥1.5, *p*-value ≤0.05) are indicated by name. **(b)** Relationship between *GREM1* gene expression and RFS in breast cancer patients using KM plotter. High and low expression were defined as above and below median. **(c)** Q-PCR analysis of *Grem1* mRNA expression in 67NR and 66cl4 cell lines in vitro (*n* = 3). Fold change is relative to *Actb* and *Tbp*. Results are shown as mean ± SEM. Student’s t-test, **0.001 < *P* < 0.01. **(d)** Representative gremlin1 immunoblot (*n* = 4) of whole lysates of the 67NR and 66cl4 cell lines without or with PTI for 6 h. (**e**) Levels of gremlin1 in conditioned medium from 67NR and 66cl4 (n = 3) measured by ELISA. Results are shown as mean ± SEM. **(f)** Q-PCR analysis of *Bmp4* mRNA expression in 67NR and 66cl4 cell lines in vitro (n = 3). Fold change is relative to *Actb* and *Tbp*. Results are shown as mean ± SEM. Student’s t-test, **0.001 < P < 0.01. **(g)** Representative BMP4 immunoblot (n = 4) of whole lysates of the 67NR and 66cl4 cell lines without or with PTI for 6 h

Cancer cells’ ability to metastasize may be affected by factors that are secreted by the cancer cells (autocrine) or by factors secreted by cells in the tumor microenvironment (TME) (paracrine). In search of factors that promote metastasis, we therefore compared the expression of genes affecting the TME in the non-metastatic 67NR and the metastatic 66cl4 cells from the 4T1 mouse model. Using RNA-sequencing (RNA-seq), we compared the transcriptomes of cells grown in culture and isolated from primary tumors in mice and we focused on transcripts encoding matrisome-associated extracellular matrix (ECM) affiliated proteins (164 genes) and secreted factors (363 genes) as defined by Naba et al. [30]. We detected 220 of these transcripts in either cell cultures or primary tumors of 66cl4 or 67NR (cut-off: 1 fragment per kilobase of mRNA per million mapped reads [FPKM]). Of these, 28 genes were significantly overexpressed in both cells and primary tumors in 66cl4 compared to 67NR (cut-off: fold-change ≥1.5, *p*-value ≤0.05) (Fig. 1a). We then analyzed if increased mRNA expression levels of any of these 28 secreted factors correlated with reduced RFS in breast cancer patients. Using KM plotter [20, 21], we found that high expression of *GREM1* correlated with RFS in all breast cancer cases (HR = 1.32 (1.18–1.47), p-value = 6.9e^− 07^) and ER-negative subtype (HR = 1.51 (1.2–1.9), p-value = 0.00037), whereas in the ER-positive subtype there was a significant correlation with RFS, but the HR was just below cut-off (HR = 1.19 (1.01–1.4), p-value = 0.035) (Fig. 1b). Of note, a poorer overall survival (OS) was also seen in the patients with the highest levels of *GREM1* mRNA in tumor biopsies (Additional file 2: Figure S1). We confirmed overexpression of *Grem1* mRNA levels in 66cl4 by quantitative PCR (Q-PCR) (Fig. 1c). Moreover, western blot and ELISA analysis showed that gremlin1 was also highly expressed on protein level and was secreted by 66cl4, but not 67NR (Fig. 1d and e). Surprisingly, *Bmp4*, which is one of the three preferred ligands that gremlin1 binds and inhibits, was also among the 28 upregulated secreted factors in the metastatic 66cl4 cells compared to the non-metastatic 67NR cells (Fig. 1a). Overexpression of *Bmp4* in 66cl4 was validated by Q-PCR (Fig. 1f) and western blot (Fig. 1g). However, besides *GREM1*, high mRNA levels of *BMP4* or any of the other 28 secreted factors that were upregulated in the metastatic 66cl4 cells did not correlate with reduced RFS (Additional file 3: Table S1). On the contrary, a reduced RFS correlated with low mRNA levels for some of these genes. We therefore wanted to study the role of gremlin1 in aggressive tumor development and metastasis more carefully.

### Genetic alterations in BMP-antagonists are common in invasive breast cancer

**Fig. 2:**
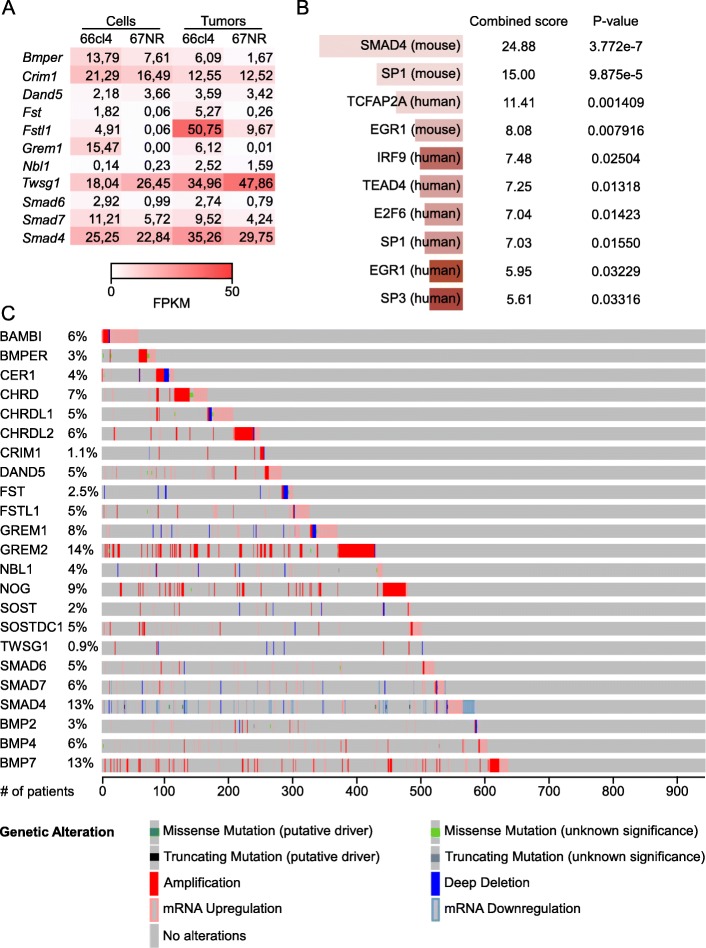
BMP-antagonists are genetically altered in a high frequency of breast cancer biopsies. (**a**) Heat-map showing the RNA-seq expression levels of 11 BMP-antagonists (cut-off: expression level ≥ 1 in either in vitro cultivated cells or tumors of 67NR and 66cl4). Values are given in fragments per kilobase of transcripts per million fragments mapped (FPKM). (**b**) Gene enrichment analysis of the 1252 genes significantly upregulated in 67NR cells and primary tumors using the Enrichr online tool (TRANSFAC and JASPAR position weight matrices). (**c**) Genetic alterations in extracellular, intracellular and transmembrane BMP-antagonists, as well as BMP2, BMP4 and BMP7 were analyzed using cBioPortal in 960 patient samples from The Cancer Genome Atlas (TCGA) provisional data set for invasive breast carcinoma

Increased gremlin1 activity has been shown to promote epithelial-to-mesenchymal transition (EMT) and maintenance of stem cell-like properties in glioma and colorectal cancer cells [9, 10, 31]. In addition, other BMP-antagonists, including Dand5 [8] and noggin [32], have been linked to tumor progression and metastasis. The RNA-seq analysis revealed that also other BMP-antagonists were expressed (> 1 FPKM) and significantly upregulated either in cell culture or primary tumors in 66cl4 compared to 67NR (Fig. 2a and Additional file 4: Table S2). Furthermore, the RNA-seq analysis showed a significant upregulation of the two inhibitory SMADs, *Smad6* and *Smad7*, in 66cl4 (Fig. 2a). Thus, BMP activity may be more repressed in the metastatic 66cl4 cells than the non-metastatic 67NR cells, both by extracellular antagonists and intracellular inhibitors. Consistently, we found increased SMAD4-related gene expression in 67NR by gene enrichment analysis (Enrichr [24, 25]) of the 1252 transcripts that were significantly upregulated in the 67NR cells and primary tumors compared with 66cl4 (Fig. 2b). To assess if our finding in the mouse model could have clinical relevance, we searched the TCGA provisional data set for invasive breast carcinoma in the online tool cBioPortal [22, 23] for gene alterations in BMP-antagonists and SMADs in invasive breast cancer patient samples. Since there is no complete list of existing BMP-antagonists, we chose to include the most recognized extracellular antagonists, as well as the two membrane-bound antagonists, *BAMBI* and *CRIM1*, in this comparison [7, 33]. Interestingly, we found genetic changes (mutations, amplifications, deletions or altered mRNA levels) in the selected genes in as much as 62% (53% if the SMADs are omitted) of the 960 samples analyzed (Fig. 2c). The data indicate that deregulation of BMP-antagonists in patients can happen via different mechanisms. For instance, *GREM2* is commonly amplified in breast cancer patients, indicating that elevated levels of *GREM2* origins from the cancer cells and not the surrounding tissue. In comparison, increased levels of *GREM1* mRNA (found in 6% of the tumor biopsies) are rarely caused by amplifications. Thus, elevated *GREM1* in breast cancer tumors may either originate from normal cells in the tumor microenvironment or from the tumor cells themselves.

We then asked if any of the extracellular antagonists besides *GREM1*, or inhibitory SMADs, could predict prognosis of breast cancer patients. In all breast cancer cases, elevated levels of *GREM1* and *SMAD6* correlated with poor prognosis (Fig. 1b and Additional file 5: Table S3). In contrast, low mRNA levels of six BMP-antagonists (*CER1, CHRD, CHRDL1, CRIM1*, *DAND5*, and *FST*) correlated with reduced RFS. Moreover, low mRNA levels of *BMPER, CHRDL1, CRIM1* and *SOSTDC1* correlated with reduced RFS in ER-positive breast cancer patients and high mRNA levels of *CRIM1*, *GREM1* and *SMAD6* correlated with reduced RFS in ER-negative breast cancer patients. In summary, these data indicate that in contrast to other BMP-antagonists, high *GREM1* expression is particularly relevant for aggressive tumor development in breast cancer patients.

### Gene co-expression network analysis identifies genes involved in collagen formation and ECM organization associated with *GREM1*

**Fig. 3:**
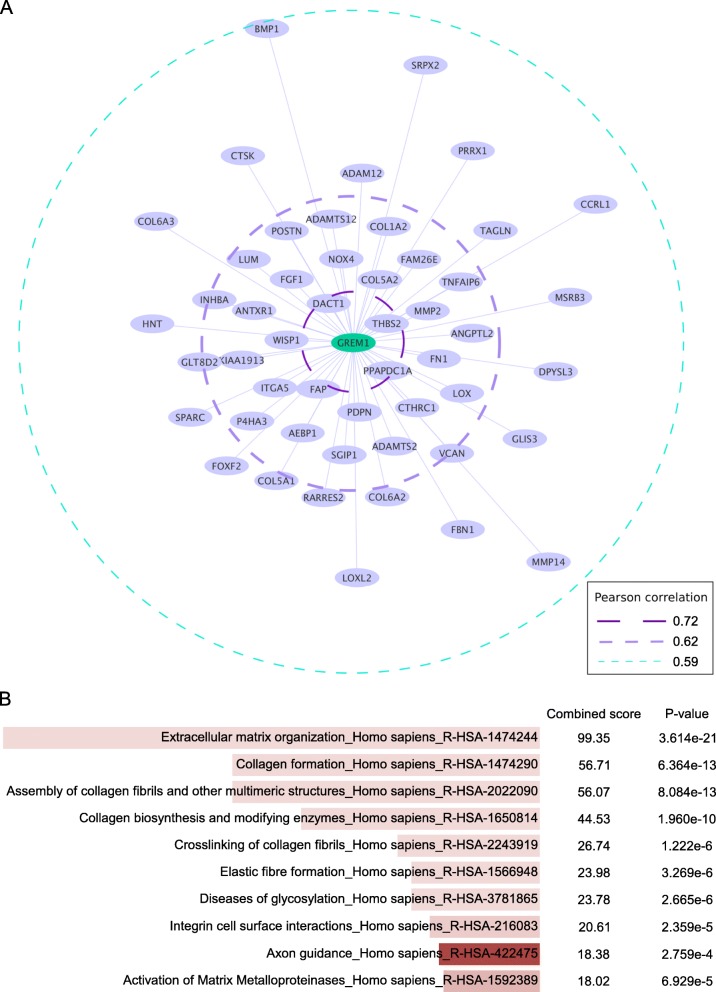
*GREM1* expression is associated with genes involved in collagen formation and extracellular matrix (ECM) organization. Co-expression analysis was done on RNA-seq data from 421 breast cancer cases downloaded from the TCGA (study by Ciriello et al., Cell, 2015) using Pearson correlation coefficient. **(a)** Depicts the 50-top scoring genes in an ego-centric network and **(b)** shows the results of the gene enrichment analysis of these genes using the Reactome pathway database

A feature of metastatic cells is the ability to undergo EMT, a process where the tumor cells lose their epithelial characteristics and gain a mesenchymal phenotype. This phenotype is associated with expression of genes that modulate ECM and allows for tumor cell motility and invasion [34]. We hypothesized that gremlin1 is involved in modulation of ECM and that *GREM1* expression correlates with expression of genes known to be involved in this process in patients. To test this, we performed a co-expression network analysis of RNA-seq data of 421 breast cancer cases from TCGA (study by Ciriello et al., Cell, 2015) [19]. The correlation values between the 16,749 genes tested and *GREM1* were in the range − 0.32 - 0.72. An ego-centric network was made by the 50 top-scoring transcripts with positive correlations [range 0.59–0.72], meaning that these genes and *GREM1* have similar expression patterns across samples (Fig. 3a). The location of the genes in the network represents the strength of association, where the ones closest to *GREM1* correlate best. Gene enrichment analysis of the same 50 top-scoring genes using the Reactome pathway database [26] demonstrated that genes involved in ECM organization, collagen formation, and collagen biosynthesis and modification were enriched (Fig. 3b). These results were confirmed by the gene co-expression search engine SEEK [27], searching 331 breast cancer data sets (Additional file 6: Table S4 and Additional file 7: Table S5). Combined, the data suggest that gremlin1 may be involved in ECM modification, which favor tumor cell migration and intravasation, leading to increased metastasis and consequently poor prognosis.

### *Grem1* depletion might impair 66cl4’s ability to form metastases in the lungs

**Fig. 4:**
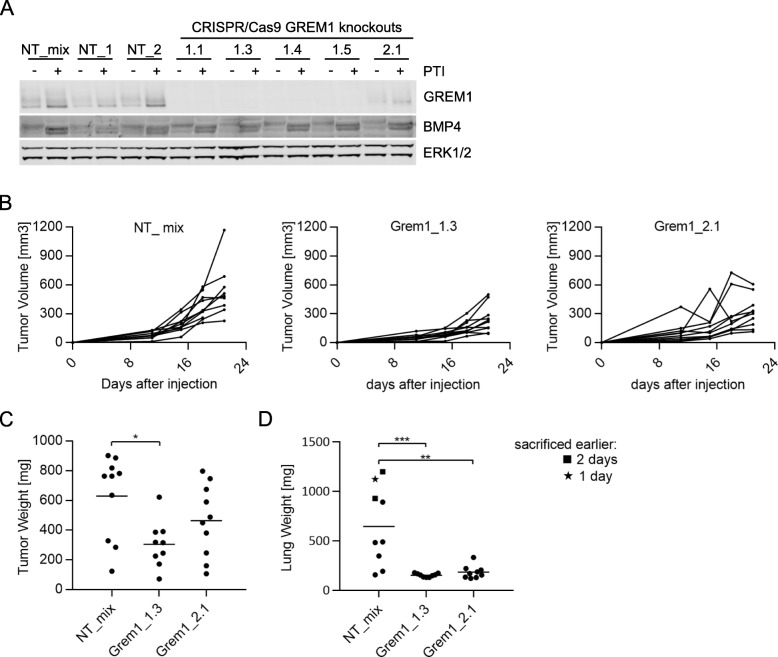
Gremlin1 depletion impairs metastases formation in the lungs**.** (**a)** Analysis of CRISPR/Cas9-mediated knockout efficiency in 66cl4. Representative immunoblot of gremlin1 of whole lysates of 66cl4 non-target controls and gremlin1 knockout clones. **(b-c)** In vivo analysis of 66cl4 non-target control and two gremlin1 knockout clones (10 mice per group). Tumor cells were injected into the fat-pad of nude mice. **(b)** The size of the primary tumors was measured regularly during the 21 days of experiment and **(c)** tumor weight was recorded after the mice had been sacrificed. The line indicates mean tumor weight. Student’s t-test, *0.01 < *P* < 0.05. **(d)** Tumor cells were injected into the tail vein of nude mice. After 15 days the mice were sacrificed and the lung weight was recorded. The line indicates mean lung weight. Student’s t-test, **0.001 < P < 0.01, *** *P* < 0.001

To determine if gremlin1 expression is important for tumor formation and progression, we used CRISPR/Cas9 to generate gremlin1 depleted 66cl4 cells. Protein expression levels were assessed by immunoblot analyses (Fig. 4a). Five clones were selected for further studies that all displayed an 80–100% reduction in gremlin1 protein levels compared to cells harboring a non-targeting (NT) guide sequence. The gremlin1 depleted clones showed no consistent change in growth rate or ability to form colonies in soft agar compared to the pooled 66cl4 NT control cells (Additional file 8: Figure S2A and B). Next, we analyzed the ability of two randomly selected clones to form primary tumors and lung metastases after injection into the fat pad and tail vein of nude mice, respectively. Unfortunately, none of the Cas9 expressing clones could form primary tumors in immunocompetent BALB/c mice, irrespective of gene editing. However, all cell lines formed tumors in immunocompromised, nude mice. Compared to control cells, the *Grem1* depleted clones Grem1_1.3 and Grem1_2.1 formed smaller primary tumors and lost their ability to metastasize to the lungs (Fig. 4b-d). In addition to the non-target cell line NT_mix, two control single cell clones (NT_1 and NT_2) were analyzed in vivo. Surprisingly, these two control sub-clones did not behave like 66cl4 wildtype or the NT_mix control cells, but rather displayed impaired primary tumor growth and metastases formation in the lungs similar to the gremlin1 depleted clones. From this, it is apparent that sub-clones originating from single 66cl4 cells have different ability to form primary and secondary tumors without any further manipulation. Nonetheless, gremlin1 is overexpressed in the metastatic 66cl4 versus the non-metastatic 67NR cells. This, and the finding that *GREM1* is frequently overexpressed in breast cancers and correlates with poor prognosis, merge on the notion that gremlin1 is important for aggressive breast cancer development.

### 66cl4 displayed increased expression of stem cell markers

**Fig. 5:**
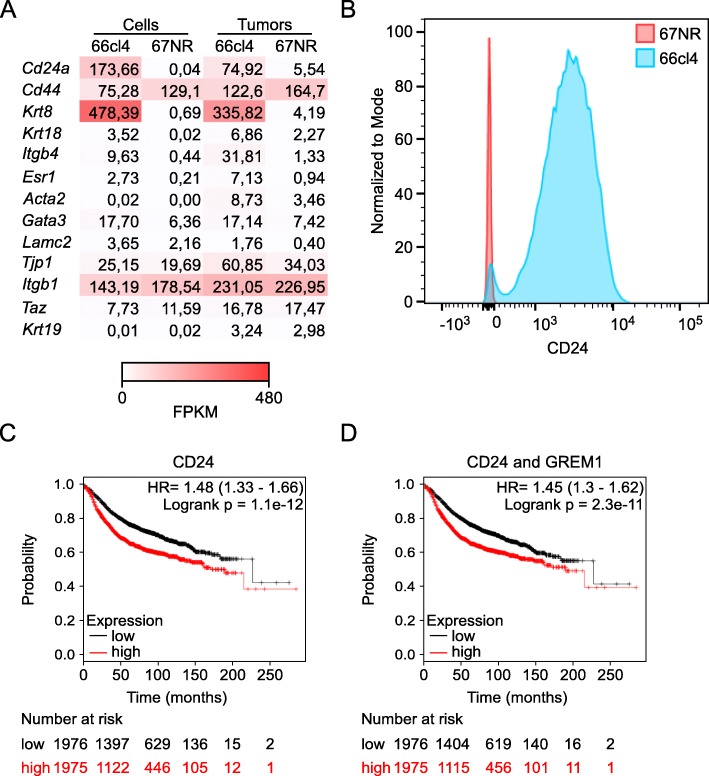
High *Grem1* levels correlates with high expression of stem cell markers in 66cl4. **(a)** Heat-map showing the RNA-Seq expression levels of 13 known stem cell markers (cut-off: expression level ≥ 1 in either cells or tumors of 67NR and 66cl4). Values are given in fragments per kilobase of transcripts per million fragments mapped (FPKM). **(b)** Representative histogram of 67NR and 66cl4 in vitro cultured cells stained with CD24 anti-mouse antibody. **(c)** Relationship between *CD24* gene expression and RFS in breast cancer patients using KM plotter. **(d)** Relationship between combined *CD24* and *GREM1* expression and RFS in breast cancer patients using KM plotter

The first step in metastasis is invasion of tumor cells into the surrounding stroma. Cancer cells of epithelial origin acquire the ability to invade as a part of the EMT, during which they reverse to an undifferentiated, stem cell-like state [12]. Since BMP-antagonists, such as gremlin1, can interfere with differentiation, we were interested in the differentiation status of the metastatic 66cl4 and the non-metastatic 67NR cells in the initial RNA-seq experiment. Analyzing the transcripts of 13 expressed stem cell markers, we found that four markers were significantly upregulated in 66cl4 cells and primary tumors (*Cd24a*, *Krt8*, *Itgb4* and *Esr1*), compared to 67NR cells and 67NR tumors (Fig. 5a and Additional file 9: Table S6). On the other side, none of the stem cell markers analyzed were significantly upregulated in 67NR compared to 66cl4. In breast cancer, a mesenchymal CD44^hi^CD24^low^ phenotype is suggested to characterize the cancer stem cells [34]. However, of the stem cell markers analyzed in 66cl4, *Cd24a* was the most clearly elevated. Flow cytometry analysis of CD24 expression on 66cl4 and 67NR in vitro cultured cells confirmed the results of the RNA-seq analysis (Fig. 5b). *Cd44*, on the other hand, showed significantly lower expression in 66cl4 compared to 67NR (Fig. 5a and Additional File 9: Table S6). Thus, the expression of these two markers indicate the opposite of expected for a classic stem cell-like metastatic phenotype (CD44^hi^CD24^low^) in 66cl4. This is consistent with previous studies showing that the metastatic potential of the 4T1 cell line is much stronger than the non-metastatic 67NR, even though 4T1 is characterized by an epithelial phenotype and the 67NR cells are more differentiated towards a mesenchymal phenotype [35]. It was also proposed that the 66cl4 cells display a poorer ability to invade and migrate in vitro compared to the 67NR cells, although 66cl4 metastasizes to lungs in vivo [36]. To get a better overview of the phenotypic profile, we used ChIP-X enrichment analysis (ChEA) in the Enrichr database to analyze the 1270 genes significantly upregulated in both 66cl4 cells and 66cl4 tumors. The analysis showed activation of several signaling pathways that are essential for stem cell maintenance, including *Oct4*, *Sox2*, *Sox9*, and *Nanog* (Additional file 10: Figure S3). Taken together, although the expression of commonly used breast cancer stem cell markers showed opposite expression than what would be expected, we found increased expression of several stem cell markers in 66cl4 compared to 67NR, supporting a metastatic stem cell-like phenotype. Since increased *GREM1* mRNA expression levels in tumors correlated with reduced RFS, we asked if high *CD24* mRNA levels also could predict poor prognosis. Similar to *GREM1*, *CD24* correlated with reduced RFS in all breast cancer cases when highly expressed (Fig. 5c). Of note, combining the expression levels for *GREM1* and *CD24* did not give any additive effect in prediction value, suggesting that their functions are closely related (Fig. 5d).

### Extravasation capacity is only marginally affected by gremlin1 depletion

**Fig. 6:**
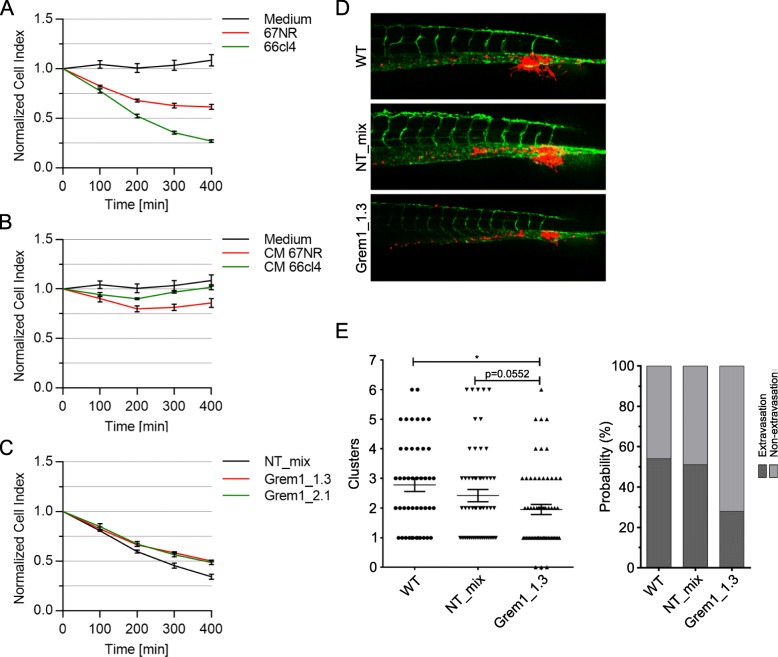
Gremlin1 depletion may affect 66cl4’s ability to extravasate**. (a-c)** Analysis of the tumor cells ability to penetrate a confluent monolayer of human umbilical vein-endothelial cells (HUVECs) using the xCELLigence Real-Time Cell Analysis (RTCA) Systems. Representative graphs depicting the change in normalized cell index after addition of tumor cells or conditioned medium. Pure growth medium was used as a control. **(a)** 67NR and 66cl4. **(b)** Conditioned medium of 67NR and 66cl4. **(c)** 66cl4 non-target control and two Grem1 knockout clones. Results are shown as mean ± SD (A-C). (**d**) Representative image of 6 dpi zebrafish larvae showing the invasion of 66cl4 wildtype cells, non-target control (NT_mix) and Grem1_1.3 (red). The vasculature is shown in green. **(e)** Quantification of invasive cluster numbers in 66cl4 injected zebrafish larvae. Probability represents the percentage of zebrafish larvae in which tumor cell extravasation was observed. Results are shown as mean ± SEM. The line indicates mean cluster number. Student’s t-test, *0.01 < P < 0.05

Since gremlin1 depletion might impair the metastatic potential of 66cl4, we next asked if 66cl4 and 67NR also display a different ability to extravasate and if gremlin1 has a role in controlling such differences. We first took advantage of an in vitro approach, where the cancer cells were scored for their ability to interfere with the integrity of a confluent layer of human umbilical vein endothelial cells (HUVECs) measured real time using impedance [37]. After seeding the HUVECs, the impedance increased until it reached a plateau. Visual inspection of the wells verified that the HUVECs had adhered and that the cultures were confluent. A small volume of culture medium containing the same number of 67NR or 66cl4 cells was added to the respective wells and impedance measured. The same volume of conditioned medium (CM) from the two cell lines was used as a negative control. Presence of 66cl4 cells caused a rapid loss in impedance (Fig. 6a). In contrast, adding the same number of 67NR cells resulted in a less prominent effect. These data suggest that the metastatic, gremlin1-expressing 66cl4 cells are better in penetrating an endothelial layer than 67NR. Interestingly, we did not see any change in impedance when adding conditioned medium of 66cl4 and 67NR to the HUVEC monolayer (Fig. 6b), indicating that changes in the endothelial cell monolayer are caused by direct interactions of the tumor cells with the HUVECs.

To test if the impaired metastatic potential of 66cl4 GREM1_1.3 and GREM1_2.1 in vivo was due to a reduced capability to extravasate, we repeated the in vitro extravasation assay with these two gremlin1 depleted cell lines. We found that both clones had a slightly reduced ability to penetrate the HUVEC monolayer compared to the NT control cells (Fig. 6c). In summary, these data suggest that the metastatic 66cl4 cells are more able to penetrate an endothelial layer than the non-metastatic 67NR cells. However, depletion of gremlin1 in 66cl4 only marginally affected the extravasation ability. As an alternative and more invasion relevant approach, we employed a xenograft model using the transgenic zebrafish line Tg(*fli1:GFP*) [16–18]. After injecting fluorescently labelled cancer cells in the duct of Cuvier (DoC) of zebrafish embryos at 48 h post fertilization, the movement of cancer cells into the avascular tail fin area, which is a measure for invasion, was documented using a fluorescent microscope. Consistent with the findings of the in vitro extravasation assay, 66cl4 wildtype cells and NT_mix cells displayed similar numbers of invasion clusters, whereas 66cl4 Grem1_1.3 formed fewer clusters in the avascular tail fin area (Fig. 6d and e).

### Co-expression of *GREM1* and BMPs does not annul the prognostic value of *GREM1* in ER-negative breast cancer patients

The finding that *Bmp4* and its antagonist *Grem1* were co-expressed in the 66cl4 metastatic cell line puzzled us. We therefore looked for co-expression of *GREM1* and its preferred BMPs, *BMP2*, *BMP4* or *BMP7*, in human breast cancer cell lines using Expression Atlas [28]. Interestingly, all the 13 breast cancer cell lines that expressed *GREM1* co-expressed at least one of the three BMPs, with *BMP4* being most commonly co-expressed (Additional file 11: Figure S4). To explore the clinical significance of these findings, we used the TCGA provisional data set for invasive breast carcinoma in cBioPortal to analyze if BMP-antagonists were co-expressed with either *BMP2*, *BMP4*, or *BMP7*. We found that many of the biopsies with a BMP amplification, also had an amplification of a gene encoding one of the BMP-antagonists (Fig. 2c). For BMP7, several amplifications were also seen without a concomitant amplification of any known antagonist.

Since deregulated BMP activity has been linked to invasion and metastasis [38], we wanted to explore the clinical significance of elevated BMP levels for survival in breast cancer patients. However, we found no correlation between high expression level of *BMP2*, *BMP4* or *BMP7* and prognosis, irrespective of sub-grouping (all breast cancer cases, ER-positive subtype, or ER-negative subtype (Table 1). Based on our findings that elevated BMP expression does not correlate with reduced RFS, whereas high *GREM1* expression is associated with poor prognosis in breast cancer, we hypothesized that combining the expression levels of BMPs and *GREM1* annuls the observed correlation of *GREM1* with RFS. Surprisingly, this was not the case. We found that in ER-negative breast cancer cases combining high expression level of *GREM1* with high expression of *BMP2*, *BMP4* or *BMP7* showed a slightly poorer prognosis compared to *GREM1* alone (Table 1). Combined, these data demonstrate that breast cancer cells may co-express BMPs and *GREM1*, and that co-expression of *BMP* and *GREM1* in breast cancer biopsies does not reduce *GREM1*’s value as a predictor of poor outcome.

**Table 1:** Combined elevated *GREM1* and *BMP* expression level improves prognostic value in ER-negative breast cancer patients. Analysis of RFS in breast cancer patients using KM plotter. High and low expression were defined as above and below median

Gene(s)	Gene ID	all BC patients		ER+ BC patients		ER- BC patients	
		HR	*p*-value	HR	*p*-value	HR	*p*-value
*GREM1*	218469_at	1.32 (1.18–1.47)	6.90E-07	1.19 (1.01–1.4)	0.035	1.51 (1.2–1.9)	0.00037
*BMP2*	205289_at	0.88 (0.79–0.98)	0.02	0.88 (0.75–1.04)	0.13	1.01 (0.81–1.26)	0.94
*BMP4*	211518_s_at	0.9 (0.81–1)	0.058	0.98 (0.83–1.16)	0.83	1.16 (0.93–1.46)	0.19
*BMP7*	209590_at	0.88 (0.79–0.98)	0.023	1.08 (0.92–1.27)	0.36	0.92 (0.73–1.15)	0.45
*BMP2/GREM1*	205289_at / 218469_at	1.33 (1.19–1.48)	3.70E-07	1.21 (1.03–1.42)	0.022	1.64 (1.31–2.07)	1.80E-05
*BMP4/GREM1*	211518_s_at / 218469_at	1.28 (1.15–1.43)	7.30E-06	1.2 (1.02–1.42)	0.026	1.63 (1.3–2.06)	2.30E-05
*BMP7/GREM1*	209590_at / 218469_at	1.29 (1.16–1.44)	4.10E-06	1.19 (1.01–1.4)	0.04	1.63 (1.3–2.06)	2.30E-05

### Increased levels of *GREM1* mRNA are associated with lack of estrogen receptor expression

**Fig. 7:**
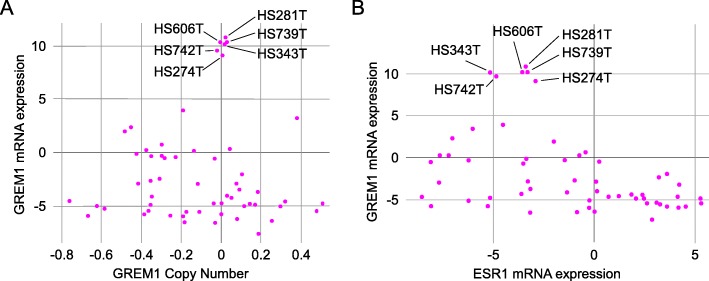
Increased levels of *GREM1* mRNA are associated with lack of estrogen receptor expression. Level of *GREM1* transcript in 60 different human breast cancer cell lines analyzed using the Cancer Cell Line Encyclopedia made available by Broad Institute. (**a**) *GREM1* transcript levels were elevated in some cell lines, but this did not correlate with *GREM1* copy number variations. (**b**) Correlation of the *GREM1* and estrogen receptor (*ERS1*) transcript levels. Transcript levels were determined using RNA-sequencing and compared to the average expression in more than 1000 human cancer cell lines for each transcript

The prognostic value of *GREM1* in ER-negative breast cancers made us ask if there could be a link between *GREM1* and ER-signaling. We therefore analyzed cell line data available from the Cancer Cell Line Encyclopedia (Broad Institute). We found that 10% (6 out of 60) breast cancer cell lines expressed elevated *GREM1* mRNA levels compared to the average of > 1000 different cell lines (Fig. 7a). The expression of *GREM1* mRNA was not caused by copy number alterations in the gene, and interestingly, all the *GREM1* expressing cell lines lacked expression of estrogen receptor, *ESR1*, mRNA (Fig. 7b). Moreover, in the TCGA provisional dataset (cBioportal) there was no overlap between breast cancer biopsies overexpressing *GREM1* (*n* = 61) or *ESR1* (*n* = 44) mRNA (data not shown), further supporting a functional link between *GREM1* expression and lack of ER-signaling.

## Discussion

Both activated BMP signaling [39–42] and elevated levels of SMAD-inhibitors [43, 44] and BMP-antagonists [8–10, 31, 32] have been linked to tumor progression and metastasis. Moreover, amplification and/or upregulation of BMPs as well as BMP-antagonists, including *NOG*, *GREM1*, *GREM2*, and *CHRD* have been reported in breast cancer [39, 45]. Yet, the simultaneous upregulation of BMPs and their antagonists, and the fact that both promote tumor aggressiveness appears contradictory. In the present study, we found that high expression of the BMP-antagonist *GREM1* correlates strongly with reduced RFS in ER-negative breast cancer.

Analyzing the metastatic 66cl4 and non-metastatic 67NR cell lines of the 4T1 mouse mammary tumor model, we found that *Grem1* was upregulated in 66cl4 cells and primary tumors. Consistent with the role of gremlin1 as an antagonist of BMP-induced differentiation, RNA-seq revealed that 66cl4 cells have elevated expression of several stem cell markers compared to 67NR. In line with Goa et al. [8], who found that overexpression of *Dand5* in the 4TO7 mouse mammary tumor cell line inhibits lung-derived BMPs and thereby promotes metastases in the lungs, we observed a reduction in lung metastases after injection of 66cl4 *Grem1* depleted cells into the tail vein of nude mice. However, high levels of *DAND5* in patients did not predict survival and may be less relevant than *GREM1* in breast cancer development. Interestingly, of the BMP-antagonists tested here, only elevated expression of *GREM1* and *CRIM1* correlated with poor prognosis in patients with ER-negative tumors.

The clinical relevance of our findings in the 4T1 mouse mammary tumor model is supported by several observations. Most importantly, high *GREM1* expression correlated with reduced relapse-free survival in ER-negative breast cancer patients. Using publicly available databases, we found that *GREM1* is also expressed on mRNA level by various human breast cancer cell lines and that *GREM1* is significantly upregulated in primary tumor biopsies of breast cancer patients compared to normal tissue samples. However, it remains unclear which cells are the source of *GREM1* in the tumor biopsies. The gene alteration data (Fig. 2c) shows that *GREM1* mRNA is elevated in approximately 6% of the breast cancer biopsies, and that amplifications are not common for this gene. It was recently shown that *GREM1* can be produced by cancer-associated fibroblasts (CAFs) in breast cancer patients and that CAFs are the main source of *GREM1* in colorectal cancer tissue [46, 47]. Of note, *GREM1* was one of eight elevated genes shown in cancer cells laser-dissected from invasive breast carcinoma patients compared with cancer cells from ductal carcinoma in situ patients [48], supporting that *GREM1* could be expressed by the transformed cancer cells themselves and associated with an invasive phenotype. Taken together, the elevated *GREM1* mRNA found in breast cancer biopsies could either come from the cancer cells themselves, or from cells infiltrating the tumor.

Among the 28 mRNAs encoding secreted proteins that were significantly upregulated in 66cl4 cells and tumors, we found not only *Grem1*, but also its ligand, *Bmp4*. Like other members of the TGF-β superfamily, BMP4 has both tumor-suppressing and tumor-promoting roles in tumor cells [40]. On one side, it has been demonstrated that BMP4 inhibits tumor cell growth. On the other side, BMP4 has been shown to increase cell migration and invasion of tumor cells. Recently, it has been described that BMP4 might regulate autophagy [49] and polarize macrophages towards an anti-inflammatory or M2-like phenotype [50]. Using web-based databases we analyzed mRNA expression levels of *GREM1* and *BMP4* in breast cancer cell lines and a breast cancer patient cohort (TCGA) and found that these two genes are also co-expressed in some tumor cell lines and primary tumors. In this way, we confirmed a possible relevance of this unexpected co-expression of both BMP4 and its antagonist gremlin1 specifically in the aggressive 66cl4 cells.

In addition to BMP4, also elevated levels of other members of the BMP-family, including BMP6 [51, 52] and BMP7 [53], have been linked to increased tumor aggressiveness. Interestingly, in contrast to *GREM1*, we found no correlation between high mRNA expression level and poor prognosis for breast cancer patients for any of the three BMPs tested. However, the correlation between *GREM1* expression levels and RFS in ER-negative breast cancer cases was not annulled when combining high expression levels of *GREM1* and *BMP2*, *BMP4* or *BMP7*. These data indicate that gremlin1 and BMP may not counteract each other with respect to cancer progression. Since gremlin1 has been shown to bind to cell surfaces [54], one could speculate that gremlin1, secreted by tumor cells or surrounding cells, binds to the surface of the transformed cancer cells and protects them from BMP-induced differentiation. On the other hand, BMPs could be further distributed in the TME and thereby induce differentiation of immune cells and stroma cells to promote tumor growth and metastasis.

In this study, we show that *GREM1* mRNA in tumor biopsies correlates with poor survival in ER-negative breast cancer patients. We propose that there may be an association between expression of *GREM1* and lack of expression of the estrogen receptor, *ESR1*, as we only found *GREM1* expression among the *ESR1* negative tumor cells. Our findings are consistent with a previous study showing that BMPs can counteract estrogen-induced cell division and that estrogen treatment led to downregulation of several BMP-receptors, including BMPR1A [55]. BMPR1A is needed for proper BMP2 and BMP4 activity and downregulating this receptor could be one way that ER-positive breast cancer cells avoid BMP activity. We speculate that ER-negative cells may develop other mechanisms to avoid BMP activity, for instance by producing their own BMP antagonists or by modulating the microenvironment to secrete factors that inhibit BMPs. We also observed that 6% of the tumor biopsies had elevated *GREM1* mRNA levels (Fig. 2c) as opposed to 10% of the breast cancer cell lines (Fig. 7). Since the source of *GREM1* in some of the tumor biopsies likely are CAFs, the proportion of cell lines that make their own *GREM1* is relatively large and this may indicate that *GREM1* gives them a growth advantage that enrich for such cells among the breast cancer cell lines.

## Conclusions

We find that gene expression of BMP-antagonists is more frequently elevated than expression of inhibitory SMADs in breast cancer biopsies. Elevated expression level of *GREM1* in tumor biopsies correlate with adverse outcome irrespective of the expression level of the BMPs in the biopsy. This suggests a dominant role for gremlin1 secretion with respect to tumor progression. The predictive value of the gene expression level for the different BMP-antagonists varies and suggests that the antagonists are functionally different in ways that must be better understood to explore these as possible drug targets against metastatic breast cancer development.

## Supplementary information

**Additional file 1:** Supplementary methods: Transcriptome analysis, Quantitative PCR, Immunoblotting, Conditioned medium, ELISA, Cell proliferation assay, Soft-agar assay, Flow cytometry, and In vitro extravasation assay using xCELLigence Real-Time Cell Analysis (RTCA) Systems.

**Additional file 2: Figure S1.** High GREM1 expression correlates with OS in breast cancer patients. Relationship between GREM1 gene expression and OS in breast cancer patients using KM plotter. High and low expression was determined using best cut off.

**Additional file 3: Table S1.** Genes encoding secreted proteins that are significantly upregulated in 66cl4 and their prognostic value in breast cancer patients. High and low expression were defined as above (HR > 1.2, p-value < 0.05) and below (HR < 0.83, p-value < 0.05) median.

**Additional file 4: Table S2.** RNA-Seq expression levels of BMP-antagonists and SMADs. Expression level ≥ 1 in either cells or tumors of 67NR and 66cl4. Values are given in fragments per kilobase of transcripts per million fragments mapped (FPKM), as well as Log2 and p-values.

**Additional file 5: Table S3.** Relationship between gene expression of BMP-antagonists and RFS in breast cancer patients. High and low expression were defined as above (HR > 1.2, p-value < 0.05) and below (HR < 0.83, p-value < 0.05) median.

**Additional file 6: Table S4.** The 50 top-scoring genes that are co-expressed with GREM1 in breast cancer. Co-expression analysis of the 50 top-scoring hits that are found co-expressed with GREM1 in a search of 331 breast cancer data sets in the SEEK database.

**Additional file 7: Table S5.** GREM1 expression is associated with genes involved in extracellular matrix (ECM) and collagen fibril organization. Gene enrichment analysis (GO Biological Process (BP) terms) of 50 top-scoring hits that co-expressed with GREM1 using the SEEK database. T, term size; A, Number of genes in the co-expressed gene set with annotations in the functional database; A&T, size of overlap between the term’s gene-set and the co-expressed gene set.

**Additional file 8: Figure S2.** In vitro analysis of CRISPR/Cas9-mediated Grem1 knockouts in 66cl4. (A) Measurement of proliferation in culture (n = 4). Results are shown as mean ± SEM. Student's t-test, *0.01 < P < 0.05, *** P < 0.001. (B) Soft-agar assay. Colony area was measured in pixels (n = 3). Results are shown as mean ± SEM.

**Additional file 9: Table S6.** RNA-Seq expression levels of 13 known stem cell markers. Expression level ≥ 1 in either cells or tumors of 67NR and 66cl4. Values are given in fragments per kilobase of transcripts per million fragments mapped (FPKM), as well as Log2 and p-values.

**Additional file 10: Figure S3.** Signaling pathways maintaining stemness are activated in 66cl4. Using CHiP-X enrichment analysis (ChEA) of the 1,270 genes significantly upregulated in both 66cl4 cells and 66cl4 tumors, we found activation of several signaling pathways that are essential for stem cell maintenance.

**Additional file 11: Figure S4.** GREM1 is co-expressed with BMPs in several human breast cancer cell lines. Co-expression analysis of GREM1 and selected BMPs (BMP2, BMP4, and BMP7) in human breast cancer cell lines using Expression atlas.

## Data Availability

The transcriptome data obtained by sequencing mRNA isolated from cells and primary breast tumors of 67NR and 66cl4 is accessible from NCBI (https://www.ncbi.nlm.nih.gov/biosample, SRA accession PRJNA577616).

## Abbreviations

BMP: Bone morphogenetic protein
ChEA: ChIP-X enrichment analysis
CM: Conditioned medium
DoC: Duct of Cuvier
Dpi: Days post implantation
ECM: Extracellular matrix
EMT: Epithelial-to-mesenchymal transition
ER: Estrogen receptor
FPKM: Fragment per kilobase of mRNA per million mapped reads
GDF: Growth and differentiation factor
HR: Hazard ratio
HUVECs: human umbilical vein endothelial cells
NT: Non-targeting
OS: Overall survival
Q-PCR: Quantitative-PCR
RFS: Relapse free survival
RNA-seq: RNA-sequencing
SD: Standard deviation
SEEK: Search-Based Exploration of Expression Compendium [Human]
SEM: Standard error of the mean
TCGA: The Cancer Genome Atlas
TGF-β: transforming growth factor-β
TME: Tumor microenvironment

## References

[1] Wakefield Lalage M., Hill Caroline S. (2013). Beyond TGFβ: roles of other TGFβ superfamily members in cancer. Nature Reviews Cancer.

[2] Colak Selcuk, ten Dijke Peter (2017). Targeting TGF-β Signaling in Cancer. Trends in Cancer.

[3] Tang Binwu, Vu Mary, Booker Timberly, Santner Steven J., Miller Fred R., Anver Miriam R., Wakefield Lalage M. (2003). TGF-β switches from tumor suppressor to prometastatic factor in a model of breast cancer progression. Journal of Clinical Investigation.

[4] Mu Y, Gudey SK, Landstrom M (2012). Non-Smad signaling pathways. Cell Tissue Res.

[5] Zhang L, Ye Y, Long X, Xiao P, Ren X, Yu J (2016). BMP signaling and its paradoxical effects in tumorigenesis and dissemination. Oncotarget.

[6] Avsian-Kretchmer O, Hsueh AJ (2004). Comparative genomic analysis of the eight-membered ring cystine knot-containing bone morphogenetic protein antagonists. Molecular endocrinology (Baltimore, Md).

[7] Walsh DW, Godson C, Brazil DP, Martin F (2010). Extracellular BMP-antagonist regulation in development and disease: tied up in knots. Trends Cell Biol.

[8] Gao H, Chakraborty G, Lee-Lim AP, Mo Q, Decker M, Vonica A, Shen R, Brogi E, Brivanlou AH, Giancotti FG (2012). The BMP inhibitor coco reactivates breast cancer cells at lung metastatic sites. Cell.

[9] Yan K, Wu Q, Yan DH, Lee CH, Rahim N, Tritschler I, DeVecchio J, Kalady MF, Hjelmeland AB, Rich JN (2014). Glioma cancer stem cells secrete Gremlin1 to promote their maintenance within the tumor hierarchy. Genes Dev.

[10] Davis H, Irshad S, Bansal M, Rafferty H, Boitsova T, Bardella C, Jaeger E, Lewis A, Freeman-Mills L, Giner FC (2015). Aberrant epithelial GREM1 expression initiates colonic tumorigenesis from cells outside the stem cell niche. Nat Med.

[11] Hanahan D, Weinberg RA (2011). Hallmarks of cancer: the next generation. Cell.

[12] Batlle E, Clevers H (2017). Cancer stem cells revisited. Nat Med.

[13] Dexter DL, Kowalski HM, Blazar BA, Fligiel Z, Vogel R, Heppner GH (1978). Heterogeneity of tumor cells from a single mouse mammary tumor. Cancer Res.

[14] Miller FR, Miller BE, Heppner GH (1983). Characterization of metastatic heterogeneity among subpopulations of a single mouse mammary tumor: heterogeneity in phenotypic stability. Invasion & metastasis.

[15] Church RH, Krishnakumar A, Urbanek A, Geschwindner S, Meneely J, Bianchi A, Basta B, Monaghan S, Elliot C, Stromstedt M (2015). Gremlin1 preferentially binds to bone morphogenetic protein-2 (BMP-2) and BMP-4 over BMP-7. The Biochemical journal.

[16] Lawson ND, Weinstein BM (2002). In vivo imaging of embryonic vascular development using transgenic zebrafish. Dev Biol.

[17] He S, Lamers GE, Beenakker JW, Cui C, Ghotra VP, Danen EH, Meijer AH, Spaink HP, Snaar-Jagalska BE (2012). Neutrophil-mediated experimental metastasis is enhanced by VEGFR inhibition in a zebrafish xenograft model. J Pathol.

[18] Ren J, Liu S, Cui C, Ten Dijke P. Invasive behavior of human breast Cancer cells in embryonic Zebrafish. Journal of visualized experiments : JoVE. 2017;122. 10.3791/55459 PMC5565102 28518096

[19] Ciriello G, Gatza ML, Beck AH, Wilkerson MD, Rhie SK, Pastore A, Zhang H, McLellan M, Yau C, Kandoth C (2015). Comprehensive molecular portraits of invasive lobular breast Cancer. Cell.

[20] Gyorffy B, Lanczky A, Eklund AC, Denkert C, Budczies J, Li Q, Szallasi Z (2010). An online survival analysis tool to rapidly assess the effect of 22,277 genes on breast cancer prognosis using microarray data of 1,809 patients. Breast Cancer Res Treat.

[21] Nagy A, Lanczky A, Menyhart O, Gyorffy B (2018). Validation of miRNA prognostic power in hepatocellular carcinoma using expression data of independent datasets. Sci Rep.

[22] Gao J., Aksoy B. A., Dogrusoz U., Dresdner G., Gross B., Sumer S. O., Sun Y., Jacobsen A., Sinha R., Larsson E., Cerami E., Sander C., Schultz N. (2013). Integrative Analysis of Complex Cancer Genomics and Clinical Profiles Using the cBioPortal. Science Signaling.

[23] Cerami E, Gao J, Dogrusoz U, Gross BE, Sumer SO, Aksoy BA, Jacobsen A, Byrne CJ, Heuer ML, Larsson E (2012). The cBio cancer genomics portal: an open platform for exploring multidimensional cancer genomics data. Cancer discovery.

[24] Chen EY, Tan CM, Kou Y, Duan Q, Wang Z, Meirelles GV, Clark NR, Ma'ayan A (2013). Enrichr: interactive and collaborative HTML5 gene list enrichment analysis tool. BMC bioinformatics.

[25] Kuleshov MV, Jones MR, Rouillard AD, Fernandez NF, Duan Q, Wang Z, Koplev S, Jenkins SL, Jagodnik KM, Lachmann A (2016). Enrichr: a comprehensive gene set enrichment analysis web server 2016 update. Nucleic Acids Res.

[26] Fabregat A, Jupe S, Matthews L, Sidiropoulos K, Gillespie M, Garapati P, Haw R, Jassal B, Korninger F, May B (2018). The Reactome pathway knowledgebase. Nucleic Acids Res.

[27] Zhu Qian, Wong Aaron K, Krishnan Arjun, Aure Miriam R, Tadych Alicja, Zhang Ran, Corney David C, Greene Casey S, Bongo Lars A, Kristensen Vessela N, Charikar Moses, Li Kai, Troyanskaya Olga G (2015). Targeted exploration and analysis of large cross-platform human transcriptomic compendia. Nature Methods.

[28] Petryszak R, Keays M, Tang YA, Fonseca NA, Barrera E, Burdett T, Fullgrabe A, Fuentes AM, Jupp S, Koskinen S (2016). Expression atlas update--an integrated database of gene and protein expression in humans, animals and plants. Nucleic Acids Res.

[29] Ghandi M, Huang FW, Jane-Valbuena J, Kryukov GV, Lo CC, McDonald ER, Barretina J, Gelfand ET, Bielski CM, Li H (2019). Next-generation characterization of the Cancer cell line encyclopedia. Nature.

[30] Naba A, Clauser KR, Hoersch S, Liu H, Carr SA, Hynes RO (2012). The matrisome: in silico definition and in vivo characterization by proteomics of normal and tumor extracellular matrices. Molecular & cellular proteomics : MCP.

[31] Karagiannis GS, Musrap N, Saraon P, Treacy A, Schaeffer DF, Kirsch R, Riddell RH, Diamandis EP (2015). Bone morphogenetic protein antagonist gremlin-1 regulates colon cancer progression. Biol Chem.

[32] Hsu MY, Rovinsky SA, Lai CY, Qasem S, Liu X, How J, Engelhardt JF, Murphy GF (2008). Aggressive melanoma cells escape from BMP7-mediated autocrine growth inhibition through coordinated Noggin upregulation. Laboratory investigation; a journal of technical methods and pathology.

[33] Wilkinson L, Kolle G, Wen D, Piper M, Scott J, Little M (2003). CRIM1 regulates the rate of processing and delivery of bone morphogenetic proteins to the cell surface. J Biol Chem.

[34] Lamouille S, Xu J, Derynck R (2014). Molecular mechanisms of epithelial-mesenchymal transition. Nat Rev Mol Cell Biol.

[35] Marsan M, Van den Eynden G, Limame R, Neven P, Hauspy J, Van Dam PA, Vergote I, Dirix LY, Vermeulen PB, Van Laere SJ (2014). A core invasiveness gene signature reflects epithelial-to-mesenchymal transition but not metastatic potential in breast cancer cell lines and tissue samples. PLoS One.

[36] Lou Y, Preobrazhenska O, Auf dem Keller U, Sutcliffe M, Barclay L, PC MD, Roskelley C, Overall CM, Dedhar S (2008). Epithelial-mesenchymal transition (EMT) is not sufficient for spontaneous murine breast cancer metastasis. Developmental dynamics : an official publication of the American Association of Anatomists.

[37] Rahim S, Uren A. A real-time electrical impedance based technique to measure invasion of endothelial cell monolayer by cancer cells. Journal of visualized experiments : JoVE. 2011;50. 10.3791/2792 PMC3169283 21490581

[38] Ren Jiang, ten Dijke Peter (2017). Bone Morphogenetic Proteins in the Initiation and Progression of Breast Cancer. Bone Morphogenetic Proteins: Systems Biology Regulators.

[39] Owens P, Pickup MW, Novitskiy SV, Giltnane JM, Gorska AE, Hopkins CR, Hong CC, Moses HL (2015). Inhibition of BMP signaling suppresses metastasis in mammary cancer. Oncogene.

[40] Ketolainen JM, Alarmo EL, Tuominen VJ, Kallioniemi A (2010). Parallel inhibition of cell growth and induction of cell migration and invasion in breast cancer cells by bone morphogenetic protein 4. Breast Cancer Res Treat.

[41] Alarmo EL, Parssinen J, Ketolainen JM, Savinainen K, Karhu R, Kallioniemi A (2009). BMP7 influences proliferation, migration, and invasion of breast cancer cells. Cancer Lett.

[42] Owens P, Polikowsky H, Pickup MW, Gorska AE, Jovanovic B, Shaw AK, Novitskiy SV, Hong CC, Moses HL (2013). Bone morphogenetic proteins stimulate mammary fibroblasts to promote mammary carcinoma cell invasion. PLoS One.

[43] de Boeck M, Cui C, Mulder AA, Jost CR, Ikeno S, Ten Dijke P (2016). Smad6 determines BMP-regulated invasive behaviour of breast cancer cells in a zebrafish xenograft model. Sci Rep.

[44] Stolfi C, Marafini I, De Simone V, Pallone F, Monteleone G (2013). The dual role of Smad7 in the control of cancer growth and metastasis. Int J Mol Sci.

[45] Tarragona M, Pavlovic M, Arnal-Estape A, Urosevic J, Morales M, Guiu M, Planet E, Gonzalez-Suarez E, Gomis RR (2012). Identification of NOG as a specific breast cancer bone metastasis-supporting gene. J Biol Chem.

[46] Dutton LR, Hoare OP, McCorry AMB, Redmond KL, Adam NE, Canamara S, Bingham V, Mullan PB, Lawler M, Dunne PD (2019). Fibroblast-derived Gremlin1 localises to epithelial cells at the base of the intestinal crypt. Oncotarget.

[47] Ren J, Smid M, Iaria J, Salvatori DCF, van Dam H, Zhu HJ, Martens JWM, Ten Dijke P (2019). Cancer-associated fibroblast-derived gremlin 1 promotes breast cancer progression. Breast cancer research : BCR.

[48] Schultz S, Bartsch H, Sotlar K, Petat-Dutter K, Bonin M, Kahlert S, Harbeck N, Vogel U, Seeger H, Fehm T (2018). Progression-specific genes identified in microdissected formalin-fixed and paraffin-embedded tissue containing matched ductal carcinoma in situ and invasive ductal breast cancers. BMC Med Genet.

[49] Deng G, Zeng S, Qu Y, Luo Q, Guo C, Yin L, Han Y, Li Y, Cai C, Fu Y (2018). BMP4 promotes hepatocellular carcinoma proliferation by autophagy activation through JNK1-mediated Bcl-2 phosphorylation. Journal of experimental & clinical cancer research : CR.

[50] Martinez VG, Rubio C, Martinez-Fernandez M, Segovia C, Lopez-Calderon F, Garin MI, Teijeira A, Munera-Maravilla E, Varas A, Sacedon R (2017). BMP4 induces M2 macrophage polarization and favors tumor progression in bladder Cancer. Clinical cancer research : an official journal of the American Association for Cancer Research.

[51] Stieglitz D, Lamm S, Braig S, Feuerer L, Kuphal S, Dietrich P, Arndt S, Echtenacher B, Hellerbrand C, Karrer S, et al. BMP6-induced modulation of the tumor micro-milieu. Oncogene. 2018. 10.1038/s41388-018-0475-x 30171260

[52] Darby S, Cross SS, Brown NJ, Hamdy FC, Robson CN (2008). BMP-6 over-expression in prostate cancer is associated with increased id-1 protein and a more invasive phenotype. J Pathol.

[53] Alarmo EL, Korhonen T, Kuukasjärvi T, Huhtala H, Holli K, Kallioniemi A (2008). Bone morphogenetic protein 7 expression associates with bone metastasis in breast carcinomas. Ann Oncol.

[54] Costello CM, Cahill E, Martin F, Gaine S, McLoughlin P (2010). Role of gremlin in the lung: development and disease. Am J Respir Cell Mol Biol.

[55] Takahashi M, Otsuka F, Miyoshi T, Otani H, Goto J, Yamashita M, Ogura T, Makino H, Doihara H (2008). Bone morphogenetic protein 6 (BMP6) and BMP7 inhibit estrogen-induced proliferation of breast cancer cells by suppressing p38 mitogen-activated protein kinase activation. J Endocrinol.

