# Supplementary material for: GREM1 is associated with metastasis and predicts poor prognosis in ER-negative breast cancer patients
Source: Cell Commun Signal. 2019 Nov 6;17:140. doi: 10.1186/s12964-019-0467-7 (PMC6836336; doi:10.1186/s12964-019-0467-7)
Supplement: Supplementary file 1 — Additional file 1: Supplementary methods: Transcriptome analysis, Quantitative PCR, Immunoblotting, Conditioned medium, ELISA, Cell proliferation assay, Soft-agar assay, Flow cytometry, and In vitro extravasation assay using xCELLigence Real-Time Cell Analysis (RTCA) Systems. [file 12964_2019_467_MOESM1_ESM.pdf]

Additional file 1

Neckmann and Wolowczyk et al. GREM1 is associated with metastasis and predicts poor prognosis in ER-negative breast cancer patients

Supplementary Methods

Transcriptome analysis

RNA was isolated from three passages of 67NR and 66cl4 cells; four and seven primary tumors of 67NR and 66cl4, respectively, and six 66cl4 lung metastases. Lung metastases were collected 2-3 weeks after removal of the primary tumors. All samples were stored in RNAlater (Qiagen, 76104) prior to RNA isolation. Tissue samples were homogenized with 1.4 mm ceramic beads from Precellys and QIAshredder (Qiagen, 79654). RNA from cells and tumors was isolated using RNeasy Plus Mini Kit (Qiagen, 74134). RNA from metastases was isolated using RNeasy Micro Kit (Qiagen, 74004). RNA-seq libraries were prepared using TruSeq Stranded mRNA kit (Illumina Inc., San Diego, CA, USA), normalized, pooled to 22 pM and subjected to clustering (by a cBot Cluster Generation System on a HiSeq2500 high output run mode flowcell (Illumina). The sequencing (2X100 cycles paired end reads) was performed on an Illumina HiSeq2500 instrument. FASTQ files were created with bcl2fastq 2.18 (Illumina). All sequencing reads of RNA-seq were mapped to the Mouse genome by splice-aware aligner Tophat2 with default settings (http://ccb.jhu.edu/software/tophat/index.shtml; version 2.0.11). Gene expression level measured in fragments per kilobase of mRNA million mapped reads (FPKM), was calculated by Cufflinks v2.1.1 using gene model annotation from UCSC release mm10. FPKM values were log2-transformed to make variation similar across orders of magnitude. The differential gene expression analyses were carried out using t-test for two-condition comparison and ANOVA for three-condition comparison followed by post hoc Tukey's honestly significant difference (HSD) test. The transcriptome data obtained by sequencing mRNA isolated from cells and primary breast tumors of 67NR and 66cl4 is accessible from NCBI (SUB6422687).

Quantitative PCR

Total RNA from cells in culture was extracted using RNeasy Mini Kit (Qiagen, 74104). RNA concentration and quality were measured by Nanodrop. cDNA was synthesized from 500 ng total RNA by QuantiTect Reverse Transcription Kit (Qiagen, 205310). cDNA was diluted 1:5. Quantitative real-time PCR was performed in parallel 25 µl reactions containing 12.5 µl 2X QuantiTect SYBR Green PCR master mix (Qiagen, 204141) and 2.5 µl 10X QuantiTect Primer Assay. Qiagen QuantiTect Primer Assays used in this study: Mm_Grem1_1_SG (QT01039983), Mm_Bmp4_1_SG (QT00111174), Mm_Actb_2_SG (QT01136772), Mm_Tbp_1_SG (QT00198443). RT-PCR was performed on the StepOne plus system (Applied Biosystems, Foster City, CA, USA) using the following cycling conditions: 95ºC for 15 min, 40 cycles of 94ºC for 15 sec, 55ºC for 30 sec and 72ºC for 30 sec. Relative gene expression levels were calculated with the 2^(-delta delta CT) method. Transcripts were normalized to *Actb* and *Tbp*.

Immunoblotting

As indicated in the results, cells were treated with Protein Transport Inhibitor (eBiosciences, 00-4980) for 6 hours and proteins harvested in urea lysis buffer containing 8 M urea (Merck Millipore, 1084870500), 0.5% (v/v) Triton X-100 (Sigma, T8787), 100 mM DTT (Sigma, 646563), Complete® protease inhibitor (Roche, 1187350001) and phosphatase inhibitor cocktail II (Sigma, P5726) and III (Sigma, P0044). Protein concentration was measured using BioRad protein assay (Bio-Rad, 500-0006). Equal amounts of proteins were run on Invitrogen NuPAGE Bis-Tris protein gels, transferred onto nitrocellulose membranes and probed with antibodies against gremlin1 (CST, 12721, 1:1000), BMP4 (Abcam, ab93939, 1:1000), and ERK1/2 (CST, 9107S, 1:2000). Proteins of interest were detected with near-infrared fluorescent (IRDye) secondary antibodies (1:10,000, Li-Cor Biosciences, 926-32211, 926-32411, 926-68070) and imaged with the Odyssey Near Infrared scanner (Li-Cor Biosciences, Lincoln, Nebraska, USA).

Conditioned medium

67NR and 66cl4 were cultured in full growth medium for three days and until they reached 80-90% confluency. To remove cell debris, the conditioned medium (CM) was centrifuged at 1500 rpm for 5 minutes and filtered through a 0.22 micron filter.

ELISA

Levels of gremlin1 in 67NR and 66cl4 conditioned medium were determined using Mouse Gremlin DuoSet ELISA (R&D Systems, #DY956). Everything was done according to the manufacturer's protocol.

Cell proliferation assay

12,000 cells were seeded in 24 well plates and counted at around 24 h, 36 h, 48 h, 60 h and 72 h. Measurements were done in triplicates and a single plate was prepared for each time point. Each of the triplicates was counted three times with the Beckman Coulter Z2 Coulter Particle Count and Size analyzer. An average cell number per well was calculated for each cell line and time point ($cells_{avg}$). The natural logarithm (ln(X)) of each $cells_{avg}$ was calculated and plotted against time (h). Growth rate (µ max [h-1]) was determined and doubling time (tD [h]) calculated: ln(2)/µ max.

Soft-agar assay

2x DMEM growth medium was prepared by dissolving 10g DMEM low glucose (Sigma Aldrich, D2902), 3.7 g sodium bicarbonate, ($NaHCO_3$), and 3.5 g glucose in milli-Q water. After sterile filtration 20% fetal calf serum (FCS, Thermo Fischer Scientific, #10270-106), 4 mM L-Glutamine (Lonza Group, De-17-605E) and 100 U/ml penicillin-streptomycin (ThermoFischer Scientific, Gibco, #15070-063) were added. PH of the final 2x DMEM was

7.4. A bottom layer of a 1:1 mix of 0.75% SeaKem® LE Agarose (LONZA, 50004) and 2x DMEM was prepared and added to each well in 6 well plates. The top layer containing a 1:1 mix of 0.36% SeaKem® LE Agarose and 2x DMEM with 10,000 cells was added to the solid bottom layer. The cells were fed twice a week with 1x DMEM and incubated at 37°C and 5% CO_2 for 12 days. Plates were stained with 0.04% crystal violet in 2% ethanol/PBS for 1 hour and washed 6 x 60 min with 1x PBS while rocking. Z-stack images of 25 beacons per well of 5 to 6 wells for each cell line were taken with the EVOS FL Auto Cell Imaging System (Invitrogen, Carlsbad, California, USA). Z-stacks were merged and analyzed with CellProfiler 2.2.0.

Flow cytometry

Anti-mouse CD16/CD32 purified antibody (Affymetrix eBioscience, #14-0161-85) was added to the cells for 15 min on ice to block unspecific binding. The cells were stained with PE anti-mouse CD24 antibody (Biolegend, #101808) and incubated for 20 min on ice protected from light. The isotype control rat IgG2a, k (Biolegend, 400508) was used for gating. The cells were washed twice and resuspended in FACS buffer (PBS with 0.1% BSA and 2mM EDTA). Immediately before running the samples, the cells were stained with Zombie Aqua™ Fixable Viability Kit (Biolegend, 423101). Data was acquired on a BD LSRII and analyzed using FlowJo software (FlowJo LLC, Oregon, USA).

***In vitro* extravasation assay using xCELLigence Real-Time Cell Analysis (RTCA) Systems**

The *in vitro* invasion assay was based on a published protocol [17]. XCELLigence E-Plates® (E-plate View 96) were coated with 0.1% gelatin for one hour at 37°C. Prior background measurement, the wells were washed once with PBS and HUVEC culturing medium (50 µl/well) was added. Subsequently, 10,000 HUVECs were added in 100 µl per well and incubated 30 minutes at room temperature to ensure uniform distribution. HUVEC growth was monitored for 48 hours in the xCELLigence RTCA System. To each well with a HUVEC monolayer, 20 µl of cancer cell suspension (8000 cancer cells/well), cancer cell conditioned medium, or full growth medium was added. Changes in impedance were recorded overnight.
