# Supplementary material for: GREM1 is associated with metastasis and predicts poor prognosis in ER-negative breast cancer patients
Source: Cell Commun Signal. 2019 Nov 6;17:140. doi: 10.1186/s12964-019-0467-7 (PMC6836336; doi:10.1186/s12964-019-0467-7)
Supplement: Supplementary file 2 — Additional file 2: Figure S1. High GREM1 expression correlates with OS in breast cancer patients. Relationship between GREM1 gene expression and OS in breast cancer patients using KM plotter. High and low expression was determined using best cut off. [file 12964_2019_467_MOESM2_ESM.pdf]

Additional file 2

Neckmann and Wolowczyk et al. GREM1 is associated with metastasis and predicts poor prognosis in ER-negative breast cancer patients

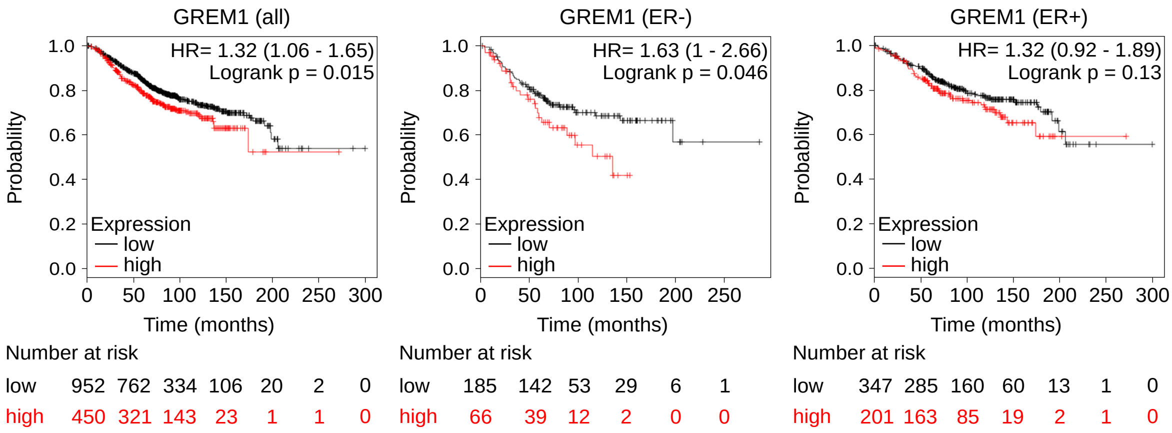


**Figure S1. High *GREM1* expression correlates with OS in breast cancer patients.** Relationship between *GREM1* gene expression and OS in breast cancer patients using KM plotter. High and low expression was determined using best cut off.
