# Supplementary material for: GREM1 is associated with metastasis and predicts poor prognosis in ER-negative breast cancer patients
Source: Cell Commun Signal. 2019 Nov 6;17:140. doi: 10.1186/s12964-019-0467-7 (PMC6836336; doi:10.1186/s12964-019-0467-7)
Supplement: Supplementary file 3 — Additional file 3: Table S1. Genes encoding secreted proteins that are significantly upregulated in 66cl4 and their prognostic value in breast cancer patients. High and low expression were defined as above (HR > 1.2, p-value < 0.05) and below (HR < 0.83, p-value < 0.05) median. [file 12964_2019_467_MOESM3_ESM.pdf]

Additional file 3

Neckmann and Wolowczyk et al. GREM1 is associated with metastasis and predicts poor prognosis in ER-negative breast cancer patients

Gene(s)	Gene ID	all BC patients		ER+ BC patients		ER- BC patients	
		HR	p-value	HR	p-value	HR	p-value
ANXA8	203074_at	0.77 (0.69 - 0.86)	3.10E-06	0.8 (0.68 - 0.95)	0.0087	0.95 (0.76 - 1.19)	0.68
AREG	205239_at	0.85 (0.76 - 0.94)	0.0024	0.92 (0.78 - 1.09)	0.34	1.15 (0.92 - 1.44)	0.22
BMP4	211518_s_at	0.9 (0.81 - 1)	0.058	0.98 (0.83 - 1.16)	0.83	1.16 (0.93 - 1.46)	0.19
CSF1	209716_at	0.64 (0.57 - 0.71)	5.6E-16	0.79 (0.67 - 0.93)	0.0039	0.79 (0.63 - 0.99)	0.044
CXCL12	209687_at	0.72 (0.65 - 0.81)	6.1E-09	0.77 (0.65 - 0.91)	0.0019	1.08 (0.86 - 1.35)	0.52
CXCL3	207850_at	0.87 (0.78 - 0.97)	0.015	0.82 (0.69 - 0.97)	0.017	0.9 (0.72 - 1.13)	0.37
FAM132B	229622_at	0.96 (0.82 - 1.12)	0.62	1.17 (0.88 - 1.57)	0.28	1.04 (0.75 - 1.45)	0.8
FGF10	231762_at	0.98 (0.84 - 1.14)	0.76	0.87 (0.65 - 1.17)	0.36	0.96 (0.69 - 1.33)	0.8
FGFBP3	238453_at	0.78 (0.67 - 0.92)	0.0022	0.98 (0.73 - 1.31)	0.88	1.01 (0.72 - 1.4)	0.96
FST	226847_at	0.68 (0.58 - 0.79)	7.30E-07	0.83 (0.62 - 1.12)	0.22	1.32 (0.94 - 1.84)	0.1
FSTL1	208782_at	0.9 (0.81 - 1.01)	0.069	1 (0.85 - 1.18)	1	1.19 (0.95 - 1.49)	0.13
GREM1	218469_at	1.32 (1.18 - 1.47)	6.90E-07	1.19 (1.01 - 1.4)	0.035	1.51 (1.2 - 1.9)	0.00037
INHBA	204926_at	1.06 (0.95 - 1.18)	0.29	1.07 (0.91 - 1.26)	0.39	1.12 (0.9 - 1.41)	0.31
INSL6	221403_s_at	0.82 (0.73 - 0.91)	2.50E-04	0.86 (0.73 - 1.02)	0.079	0.91 (0.73 - 1.14)	0.41
LGALS8	210731_s_at	0.69 (0.62 - 0.77)	2.9E-11	1.11 (0.94 - 1.3)	0.23	0.87 (0.69 - 1.09)	0.22
MEGF6	226869_at	0.79 (0.68 - 0.92)	0.0026	1 (0.75 - 1.34)	1	1.06 (0.76 - 1.48)	0.72
PDGFB	204200_s_at	1.04 (0.93 - 1.16)	0.46	1.13 (0.96 - 1.33)	0.15	1.06 (0.84 - 1.33)	0.62
PRL2C2	not found						
PRl2C3<br>PRL2C4	not found						
S100A1	205334_at	0.87 (0.78 - 0.97)	0.016	0.88 (0.75 - 1.04)	0.12	0.87 (0.7 - 1.1)	0.24
S100A13	202598_at	0.94 (0.84 - 1.05)	0.25	0.97 (0.82 - 1.14)	0.7	1.04 (0.83 - 1.31)	0.7
S100A6	217728_at	1.07 (0.96 - 1.2)	0.2	0.97 (0.83 - 1.15)	0.75	1.02 (0.81 - 1.27)	0.89
SEMA3A	244163_at	0.94 (0.81 - 1.1)	0.45	1.09 (0.82 - 1.46)	0.54	0.9 (0.64 - 1.25)	0.52
SEMA3E	206941_x_at	0.74 (0.66 - 0.83)	6.60E-08	0.68 (0.58 - 0.81)	5.90E-06	1.12 (0.89 - 1.4)	0.34
TGFB1	203084_at	1.16 (1.04 - 1.3)	0.0069	1 (0.85 - 1.18)	1	1.02 (0.81 - 1.27)	0.89
TNFSF9	206907_at	0.71 (0.64 - 0.79)	1.1E-09	0.95 (0.81 - 1.12)	0.54	0.76 (0.6 - 0.95)	0.017
VEGFC	209946_at	0.97 (0.87 - 1.08)	0.53	1.09 (0.93 - 1.29)	0.28	1.2 (0.95 - 1.5)	0.12
WNT7B	238105_x_at	1.14 (0.98 - 1.34)	0.087	0.81 (0.61 - 1.08)	0.16	1.03 (0.74 - 1.44)	0.84

Table S1. Genes encoding secreted proteins that are significantly upregulated in 66cl4 and their prognostic value in breast cancer patients. High and low expression were defined as above (HR > 1.2, p-value < 0.05) and below (HR < 0.83, p-value < 0.05) median.
