# Supplementary material for: GREM1 is associated with metastasis and predicts poor prognosis in ER-negative breast cancer patients
Source: Cell Commun Signal. 2019 Nov 6;17:140. doi: 10.1186/s12964-019-0467-7 (PMC6836336; doi:10.1186/s12964-019-0467-7)
Supplement: Supplementary file 4 — Additional file 4: Table S2. RNA-Seq expression levels of BMP-antagonists and SMADs. Expression level ≥ 1 in either cells or tumors of 67NR and 66cl4. Values are given in fragments per kilobase of transcripts per million fragments mapped (FPKM), as well as Log2 and p-values. [file 12964_2019_467_MOESM4_ESM.pdf]

Additional file 4

Neckmann and Wolowczyk et al. GREM1 is associated with metastasis and predicts poor prognosis in ER-negative breast cancer patients

		Cells				Tumors			
**Gene**	**Ensembl_id**	**66cl4**	**67NR**	**Log2**	**p-value**	**66cl4**	**67NR**	**Log2**	**p-value**
Bmper	ENSMUSG00000031963	13.7931	7.61063	0.86	0.000711895	6.08651	1.67397	1.86	1.27E-05
Crim1	ENSMUSG00000024074	21.2936	16.4902	0.37	0.140032903	12.5486	12.5226	0	0.711527403
Dand5	ENSMUSG00000053226	2.18301	3.658	-0.74	0.003151156	3.58794	3.42035	0.07	0.528880971
Fst	ENSMUSG00000021765	1.81829	0.0565305	5.01	0.004846442	5.26952	0.262559	4.33	1.90E-06
Fstl1	ENSMUSG00000022816	4.90506	0.06219	6.3	0.001721338	50.7506	9.67127	2.39	0.005137758
Grem1	ENSMUSG00000074934	15.4653	0.0040198	11.91	0.001015642	6.1211	0.0115512	9.05	1.55E-09
Nbl1	ENSMUSG00000041120	0.140468	0.230801	-0.72	0.148289133	2.5222	1.5878	0.67	0.158286831
Twsg1	ENSMUSG00000024098	18.0357	26.4537	-0.55	0.00133084	34.9632	47.8573	-0.45	0.001244606
Smad6	ENSMUSG00000036867	2.91908	0.990316	1.56	0.000617285	2.73827	0.785445	1.8	4.81E-06
Smad7	ENSMUSG00000025880	11.2136	5.72204	0.97	0.006683836	9.52152	4.24321	1.17	4.06E-06
Smad4	ENSMUSG00000024515	25.2481	22.8411	0.14	0.055946754	35.2626	29.7495	0.25	0.021002521

Table S2. RNA-Seq expression levels of BMP-antagonists and SMADs. Expression level ≥ 1 in either cells or tumors of 67NR and 66cl4. Values are given in fragments per kilobase of transcripts per million fragments mapped (FPKM), as well as Log2 and p-values.
