# Supplementary material for: GREM1 is associated with metastasis and predicts poor prognosis in ER-negative breast cancer patients
Source: Cell Commun Signal. 2019 Nov 6;17:140. doi: 10.1186/s12964-019-0467-7 (PMC6836336; doi:10.1186/s12964-019-0467-7)
Supplement: Supplementary file 5 — Additional file 5: Table S3. Relationship between gene expression of BMP-antagonists and RFS in breast cancer patients. High and low expression were defined as above (HR > 1.2, p-value < 0.05) and below (HR < 0.83, p-value < 0.05) median. [file 12964_2019_467_MOESM5_ESM.pdf]

Additional file 5

Neckmann and Wolowczyk et al. GREM1 is associated with metastasis and predicts poor prognosis in ER-negative breast cancer patients

Gene	Gene ID	all BC patients		ER+ BC patients		ER- BC patients	
		HR	p-value	HR	p-value	HR	p-value
BAMBI	203304_at	1.06 (0.95 - 1.18)	0.3	1.02 (0.86 - 1.2)	0.85	1.06 (0.85 - 1.33)	0.61
BMPER	241986_at	0.88 (0.75 - 1.02)	0.097	0.73 (0.54 - 0.98)	0.034	1.21 (0.87 -1.69)	0.25
CER1	221378_at	0.76 (0.68 - 0.85)	6.80E-07	1.07 (0.91 - 1.26)	0.44	0.94 (0.75 - 1.18)	0.58
CHRD	211248_s_at	0.72 (0.65 - 0.81)	5.20E-09	0.93 (0.79 - 1.09)	0.38	1.04 (0.83 - 1.31)	0.71
CHRDL1	209763_at	0.73 (0.66 - 0.82)	2.40E-08	0.8 (0.68 - 0.95)	0.0085	0.88 (0.7 - 1.1)	0.2686
CHRDL2	223987_at	0.88 (0.75 - 1.02)	0.0922	0.85 (0.64 - 1.14)	0.277	0.7 (0.5 - 0.98)	0.035
CRIM1	202552_s_at	0.75 (0.67 - 0.84)	2.50E-07	0.72 (0.61 - 0.85)	7.20E-05	1.34 (1.07 - 1.68)	0.011
DAND5	1562772_a_at	0.71 (0.6 - 0.83)	1.20E-05	0.93 (0.7 - 1.24)	0.63	1.17 (0.84 - 1.63)	0.35
FST	226847_at	0.68 (0.58 - 0.79)	7.30E-07	0.83 (0.82 - 1.12)	0.22	1.32 (0.94 - 1.84)	0.1
FSTL1	208782_at	0.9 (0.81 - 1.01)	0.069	1 (0.85 - 1.18)	1	1.19 (0.95 - 1.49)	0.13
GREM1	218469_at	1.32 (1.18 - 1.47)	6.90E-07	1.19 (1.01 - 1.4)	0.035	1.51 (1.2 - 1.9)	0.00037
GREM2	240509_s_at	0.86 (0.73 - 1)	0.05	0.98 (0.73 - 1.31)	0.88	0.84 (0.61 - 1.17)	0.31
NBL1	201621_at	0.86 (0.77 - 0.95)	0.0048	0.91 (0.78 - 1.08)	0.28	1.25 (0.99 - 1.56)	0.057
NOG	231798_at	0.91 (0.78 - 1.06)	0.22	0.95 (0.71 - 1.27)	0.74	1 (0.72 - 1.39)	1
SOSTDC1	213456_at	1 (0.9 - 1.12)	0.94	0.8 (0.68 - 0.94)	0.0064	0.95 (0.75 - 1.18)	0.62
SOST	223869_at	0.94 (0.8 - 1.09)	0.4	0.93 (0.7 - 1.24)	0.63	0.84 (0.6 - 1.17)	0.31
TWSG1	225406_at	0.85 (0.73 - 0.99)	0.042	0.77 (0.57 - 1.03)	0.072	1.18 (0.85 - 1.65)	0.32
SMAD6	207069_s_at	1.29 (1.15 - 1.44)	5.70E-06	1.17 (1 - 1.38)	0.054	1.65 (1.31 - 2.08)	1.50E-05
SMAD7	204790_at	0.92 (0.82 - 1.02)	0.12	1.1 (0.93 - 1.3)	0.25	1.04 (0.83 - 1.31)	0.71
SMAD4	235725_at	0.65 (0.55 - 0.76)	4.10E-08	0.78 (0.58 - 1.04)	0.086	1.25 (0.89 - 1.74)	0.19

Table S3. Relationship between gene expression of BMP-antagonists and RFS in breast cancer patients. High and low expression were defined as above (HR > 1.2, p-value < 0.05) and below (HR < 0.83, p-value < 0.05) median.
