# Supplementary material for: GREM1 is associated with metastasis and predicts poor prognosis in ER-negative breast cancer patients
Source: Cell Commun Signal. 2019 Nov 6;17:140. doi: 10.1186/s12964-019-0467-7 (PMC6836336; doi:10.1186/s12964-019-0467-7)
Supplement: Supplementary file 6 — Additional file 6: Table S4. The 50 top-scoring genes that are co-expressed with GREM1 in breast cancer. Co-expression analysis of the 50 top-scoring hits that are found co-expressed with GREM1 in a search of 331 breast cancer data sets in the SEEK database. [file 12964_2019_467_MOESM6_ESM.pdf]

Additional file 6

Neckmann and Wolowczyk et al. GREM1 is associated with metastasis and predicts poor prognosis in ER-negative breast cancer patients

Rank	Gene	Entrez ID	Coexpression Score	P-Value	Description
1	DACT1	51339	1.3157	0.0000	dapper, antagonist of beta-catenin, homolog 1 (Xenopus laevis)
2	FAP	2191	1.3019	0.0000	fibroblast activation protein, alpha
3	THBS2	7058	1.2995	0.0000	thrombospondin 2
4	COL6A3	1293	1.274	0.0001	collagen, type VI, alpha 3
5	NID2	22795	1.2728	0.0000	nidogen 2 (osteonidogen)
6	COL1A2	1278	1.2653	0.0010	collagen, type I, alpha 2
7	COL5A2	1290	1.2598	0.0005	collagen, type V, alpha 2
8	ADAM12	8038	1.2558	0.0006	ADAM metallopeptidase domain 12
9	MMP2	4313	1.2518	0.0010	matrix metallopeptidase 2 (gelatinase A, 72kDa gelatinase, 72kDa type IV collagenase)
10	COL3A1	1281	1.2481	0.0009	collagen, type III, alpha 1
11	FBN1	2200	1.2386	0.0017	fibrillin 1
12	DPYSL3	1809	1.2108	0.0002	dihydropyrimidinase-like 3
13	VCAN	1462	1.2084	0.0000	versican
14	POSTN	10631	1.2056	0.0000	periostin, osteoblast specific factor
15	SPOCK1	6695	1.202	0.0001	sparc/osteonectin, cwcv and kazal-like domains proteoglycan (testican) 1
16	PRRX1	5396	1.2002	0.0005	paired related homeobox 1
17	LUM	4060	1.1973	0.0006	lumican
18	SPARC	6678	1.1968	0.0036	secreted protein, acidic, cysteine-rich (osteonectin)
19	TNFAIP6	7130	1.1964	0.0010	tumor necrosis factor, alpha-induced protein 6
20	COL10A1	1300	1.1817	0.0001	collagen, type X, alpha 1
21	FN1	2335	1.1797	0.0006	fibronectin 1
22	COL1A1	1277	1.1775	0.0029	collagen, type I, alpha 1
23	PPAPDC1A	196051	1.1755	0.0001	phosphatidic acid phosphatase type 2 domain containing 1A
24	COL11A1	1301	1.1723	0.0003	collagen, type XI, alpha 1
25	INHBA	3624	1.1711	0.0006	inhibin, beta A
26	CDH11	1009	1.1683	0.0026	cadherin 11, type 2, OB-cadherin (osteoblast)
27	DKK3	27122	1.1636	0.0020	dickkopf 3 homolog (Xenopus laevis)
28	COL5A1	1289	1.1563	0.0058	collagen, type V, alpha 1
29	FSTL1	11167	1.1451	0.0058	follistatin-like 1
30	FNDC1	84624	1.1448	0.0000	fibronectin type III domain containing 1
31	DCN	1634	1.1444	0.0102	decorin
32	NID1	4811	1.1344	0.0010	nidogen 1
33	THY1	7070	1.1327	0.0040	Thy-1 cell surface antigen
34	NOX4	50507	1.1298	0.0001	NADPH oxidase 4
35	TMEM200A	114801	1.1234	0.0000	transmembrane protein 200A
36	CTHRC1	115908	1.1206	0.0004	collagen triple helix repeat containing 1
37	GLT8D2	83468	1.1194	0.0049	glycosyltransferase 8 domain containing 2
38	LOX	4015	1.1174	0.0013	lysyl oxidase

39	LRRC15	131578	1.1112	0.0016	leucine rich repeat containing 15
40	SULF1	23213	1.1101	0.0024	sulfatase 1
41	AEBP1	165	1.1082	0.0032	AE binding protein 1
42	MFAP5	8076	1.0935	0.0004	microfibrillar associated protein 5
43	PDPN	10630	1.0932	0.0004	podoplanin
44	ASPN	54829	1.0898	0.0001	asporin
45	COL8A2	1296	1.0815	0.0007	collagen, type VIII, alpha 2
46	TSHZ3	57616	1.0632	0.0016	teashirt zinc finger homeobox 3
47	BNC2	54796	1.0515	0.0029	basonuclin 2
48	CTSK	1513	1.0478	0.0035	cathepsin K
49	EFEMP2	30008	1.042	0.0171	EGF containing fibulin-like extracellular matrix protein 2
50	PDGFRB	5159	1.0417	0.0083	platelet-derived growth factor receptor, beta polypeptide

Table S4. The 50 top-scoring genes that are co-expressed with *GREM1* in breast cancer. Co-expression analysis of the 50 top-scoring hits that are found co-expressed with *GREM1* in a search of 331 breast cancer data sets in the SEEK database.
