# Supplementary material for: GREM1 is associated with metastasis and predicts poor prognosis in ER-negative breast cancer patients
Source: Cell Commun Signal. 2019 Nov 6;17:140. doi: 10.1186/s12964-019-0467-7 (PMC6836336; doi:10.1186/s12964-019-0467-7)
Supplement: Supplementary file 7 — Additional file 7: Table S5. GREM1 expression is associated with genes involved in extracellular matrix (ECM) and collagen fibril organization. Gene enrichment analysis (GO Biological Process (BP) terms) of 50 top-scoring hits that co-expressed with GREM1 using the SEEK database. T, term size; A, Number of genes in the co-expressed gene set with annotations in the functional database; A&T, size of overlap between the term’s gene-set and the co-expressed gene set. [file 12964_2019_467_MOESM7_ESM.pdf]

Additional file 7

Neckmann and Wolowczyk et al. GREM1 is associated with metastasis and predicts poor prognosis in ER-negative breast cancer patients

Term	p-value	q-value	T	A	T&A
extracellular matrix organization	2E-22	1.6E-21	221	39	21
extracellular matrix disassembly	6.9E-15	2.7E-13	82	39	12
collagen catabolic process	8.5E-15	4.3E-13	62	39	11
collagen fibril organization	2.3E-13	4.5E-11	16	39	7
skeletal system development	1.8E-05	0.0004	140	39	7
transmembrane receptor protein serine threonine kinase signaling pathway	0.00011	0.00187	179	39	7
skin development	0.00012	0.003	128	39	6
collagen biosynthetic process	0.00012	0.00643	15	39	3
organ morphogenesis	0.00229	0.0354	204	39	6
odontogenesis	0.00229	0.0481	30	39	3
sulfur compound catabolic process	0.00229	0.0782	36	39	3
cell adhesion	0.0148	0.0981	472	39	8
cell morphogenesis involved in differentiation	0.0148	0.114	373	39	7
cell migration	0.0185	0.118	496	39	8
dermatan sulfate biosynthetic process	0.0185	0.124	11	39	2
positive regulation of Wnt receptor signaling pathway	0.0185	0.124	47	39	3
regulation of transmembrane receptor protein serine threonine kinase signaling pathway	0.0185	0.124	107	39	4
sulfur compound metabolic process	0.0185	0.131	194	39	5
peptide cross linking	0.0185	0.131	13	39	2
cardiovascular system development	0.0185	0.131	301	39	6
platelet activation	0.0185	0.132	204	39	5
regulation of Wnt receptor signaling pathway	0.0185	0.138	123	39	4
neurogenesis	0.019	0.14	429	39	7
eye morphogenesis	0.019	0.14	15	39	2
proteoglycan metabolic process	0.019	0.219	68	39	3
neuron projection development	0.0245	0.222	349	39	6

Table S5. *GREM1* expression is associated with genes involved in extracellular matrix (ECM) and collagen fibril organization. Gene enrichment analysis (GO Biological Process (BP) terms) of 50 top-scoring hits that co-expressed with *GREM1* using the SEEK database. T, term size; A, Number of genes in the co-expressed gene set with annotations in the functional database; A&T, size of overlap between the term's gene-set and the co-expressed gene set.
