# Supplementary material for: GREM1 is associated with metastasis and predicts poor prognosis in ER-negative breast cancer patients
Source: Cell Commun Signal. 2019 Nov 6;17:140. doi: 10.1186/s12964-019-0467-7 (PMC6836336; doi:10.1186/s12964-019-0467-7)
Supplement: Supplementary file 8 — Additional file 8: Figure S2. In vitro analysis of CRISPR/Cas9-mediated Grem1 knockouts in 66cl4. (A) Measurement of proliferation in culture (n = 4). Results are shown as mean ± SEM. Student's t-test, *0.01 < P < 0.05, *** P < 0.001. (B) Soft-agar assay. Colony area was measured in pixels (n = 3). Results are shown as mean ± SEM. [file 12964_2019_467_MOESM8_ESM.pdf]

Additional file 8

Neckmann and Wolowczyk et al. GREM1 is associated with metastasis and predicts poor prognosis in ER-negative breast cancer patients

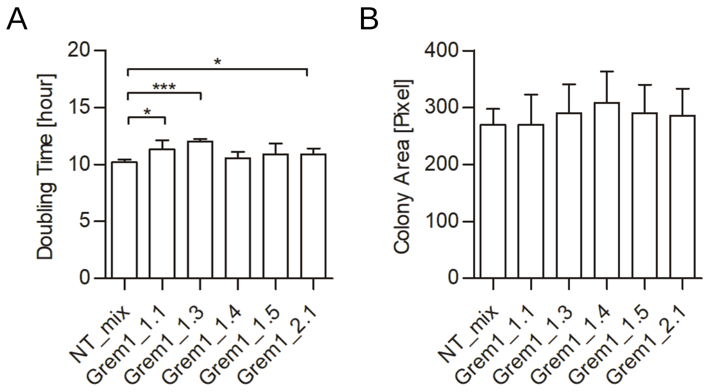

Figure S2. In vitro analysis of CRISPR/Cas9-mediated Grem1 knockouts in 66cl4.
(A) Measurement of proliferation in culture (n = 4). Results are shown as mean ± SEM. Student's t-test, *0.01 < P < 0.05, *** P < 0.001. **(B)** Soft-agar assay. Colony area was measured in pixels (n = 3). Results are shown as mean ± SEM.
