# Supplementary material for: GREM1 is associated with metastasis and predicts poor prognosis in ER-negative breast cancer patients
Source: Cell Commun Signal. 2019 Nov 6;17:140. doi: 10.1186/s12964-019-0467-7 (PMC6836336; doi:10.1186/s12964-019-0467-7)
Supplement: Supplementary file 9 — Additional file 9: Table S6. RNA-Seq expression levels of 13 known stem cell markers. Expression level ≥ 1 in either cells or tumors of 67NR and 66cl4. Values are given in fragments per kilobase of transcripts per million fragments mapped (FPKM), as well as Log2 and p-values. [file 12964_2019_467_MOESM9_ESM.pdf]

Additional file 9

Neckmann and Wolowczyk et al. GREM1 is associated with metastasis and predicts poor prognosis in ER-negative breast cancer patients

		Cells				Tumors			
**Gene**	**Ensembl_id**	**66cl4**	**67NR**	**Log2**	**p-value**	**66cl4**	**67NR**	**Log2**	**p-value**
Cd24a	ENSMUSG00000047139	173.663	0.0417377	12.02	0.000311796	74.9169	5.54322	3.76	4.74E-05
Cd44	ENSMUSG00000005087	75.2829	129.109	-0.78	0.001400073	122.622	164.682	-0.43	0.0229546
Krt8	ENSMUSG00000049382	478.391	0.690617	9.44	2.40E-07	335.816	4.18793	6.33	0.000254
Krt18	ENSMUSG00000023043	3.51505	0.0248805	7.14	0.002313837	6.86341	2.26636	1.6	0.2918238
Itgb4	ENSMUSG00000020758	9.62822	0.439601	4.45	6.04E-06	31.8102	1.32976	4.58	7.92E-06
Esr1	ENSMUSG00000019768	2.73238	0.20708	3.72	0.007083623	7.12857	0.943692	2.92	1.36E-07
Acta2	ENSMUSG00000035783	0.0159818	0.0024338	2.72	0.268070771	8.72886	3.4559	1.34	0.0064604
Gata3	ENSMUSG00000015619	17.6993	6.35715	1.48	3.12E-05	17.1415	7.41823	1.21	2.18E-05
Lamc2	ENSMUSG00000026479	3.64965	2.16038	0.76	0.009223553	1.75698	0.404321	2.12	9.71E-05
Tjp1	ENSMUSG00000030516	25.1525	19.6892	0.35	0.021408713	60.8534	34.0283	0.84	0.0014656
Itgb1	ENSMUSG00000025809	143.186	178.541	-0.32	0.059519315	231.045	226.948	0.03	0.3128417
Taz	ENSMUSG00000009995	7.72876	11.5938	-0.59	0.000666401	16.7796	17.4729	-0.06	0.7515521
Krt19	ENSMUSG00000020911	0.0092546	0.0150448	-0.7	0.773530449	3.23897	2.97902	0.12	0.9426575

Table S6. RNA-Seq expression levels of 13 known stem cell markers. Expression level ≥ 1 in either cells or tumors of 67NR and 66cl4. Values are given in fragments per kilobase of transcripts per million fragments mapped (FPKM), as well as Log2 and p-values.
