# Supplementary material for: GREM1 is associated with metastasis and predicts poor prognosis in ER-negative breast cancer patients
Source: Cell Commun Signal. 2019 Nov 6;17:140. doi: 10.1186/s12964-019-0467-7 (PMC6836336; doi:10.1186/s12964-019-0467-7)
Supplement: Supplementary file 10 — Additional file 10: Figure S3. Signaling pathways maintaining stemness are activated in 66cl4. Using CHiP-X enrichment analysis (ChEA) of the 1,270 genes significantly upregulated in both 66cl4 cells and 66cl4 tumors, we found activation of several signaling pathways that are essential for stem cell maintenance. [file 12964_2019_467_MOESM10_ESM.pdf]

Additional file 10

Neckmann and Wolowczyk et al. GREM1 is associated with metastasis and predicts poor prognosis in ER-negative breast cancer patients

	Combined score	P-value
MYB_21317192_ChIP-Seq_ERMYB_Mouse	38.44	7.540e-9
NFE2L2_20460467_ChIP-Seq_MEFs_Mouse	35.12	7.751e-9
NRF2_20460467_ChIP-Seq_MEFs_Mouse	35.01	7.751e-9
CHD7_19251738_ChIP-ChIP_MESCs_Mouse	30.20	8.289e-5
SMC1_22415368_ChIP-Seq_MEFs_Mouse	20.75	7.660e-7
TCF3_18692474_ChIP-Seq_MEFs_Mouse	20.27	2.597e-4
SOX2_27498859_Chip-Seq_STOMACH_Mouse	19.54	1.819e-6
NANOG_18692474_ChIP-Seq_MEFs_Mouse	18.39	1.515e-5
SOX2_20726797_ChIP-Seq_SW620_Human	17.53	7.092e-5
ESR1_21235772_ChIP-Seq_MCF-7_Human	16.97	0.002854
E2F1_18555785_ChIP-Seq_MESCs_Mouse	15.99	2.237e-6
ZNF217_24962896_ChIP-Seq_MCF-7_Human	15.62	1.433e-4
SOX2_18358816_ChIP-ChIP_MESCs_Mouse	15.43	0.001745
SOX2_18692474_ChIP-Seq_MEFs_Mouse	14.93	1.032e-4
NUCKS1_24931609_ChIP-Seq_HEPATOCYTES_Mouse	14.58	0.001022
OCT4_18692474_ChIP-Seq_MEFs_Mouse	14.03	7.402e-5
TCF7_22412390_ChIP-Seq_EML_Mouse	13.67	9.106e-5
ZFP281_18358816_ChIP-ChIP_MESCs_Mouse	13.33	0.007472
SOX9_24532713_ChIP-Seq_HFSC_Mouse	13.25	1.983e-4
SFPI1_20887958_ChIP-Seq_HPC-7_Mouse	12.88	3.761e-5
BCL3_23251550_ChIP-Seq_MUSCLE_Mouse	12.79	9.174.-4
PPARG_19300518_ChIP-PET_3T3-L1_Mouse	12.69	0.01477
WT1_25993318_ChIP-Seq_PODOCYTE_Human	12.48	7.821e-6
TFAP2C_20629094_ChIP-Seq_MCF-7_Human	11.58	0.001195
THRA_23701648_ChIP-Seq_CEREBELLUM_Mouse	11.12	0.01982
STAT3_24763339_ChIP-Seq_IMN-ESCs_Mouse	11.08	4.735e-4
IRF8_27001747_Chip-Seq_BMDM_Mouse	11.06	3.581e-4
GFI1B_20887958_ChIP-Seq_HPC-7_Mouse	10.61	6.447e-4
CEBPD_21427703_ChIP-Seq_3T3-L1_Mouse	10.54	7.074e-4
CEBPB_21427703_ChIP-Seq_3T3-L1_Mouse	10.44	4,955e-4

Figure S3. Signaling pathways maintaining stemness are activated in 66cl4. Using CHiP-X enrichment analysis (ChEA) of the 1,270 genes significantly upregulated in both 66cl4 cells and 66cl4 tumors, we found activation of several signaling pathways that are essential for stem cell maintenance.
