# Supplementary material for: GREM1 is associated with metastasis and predicts poor prognosis in ER-negative breast cancer patients
Source: Cell Commun Signal. 2019 Nov 6;17:140. doi: 10.1186/s12964-019-0467-7 (PMC6836336; doi:10.1186/s12964-019-0467-7)
Supplement: Supplementary file 11 — Additional file 11: Figure S4. GREM1 is co-expressed with BMPs in several human breast cancer cell lines. Co-expression analysis of GREM1 and selected BMPs (BMP2, BMP4, and BMP7) in human breast cancer cell lines using Expression atlas. [file 12964_2019_467_MOESM11_ESM.pdf]

Additional file 11

Neckmann and Wolowczyk et al. GREM1 is associated with metastasis and predicts poor prognosis in ER-negative breast cancer patients

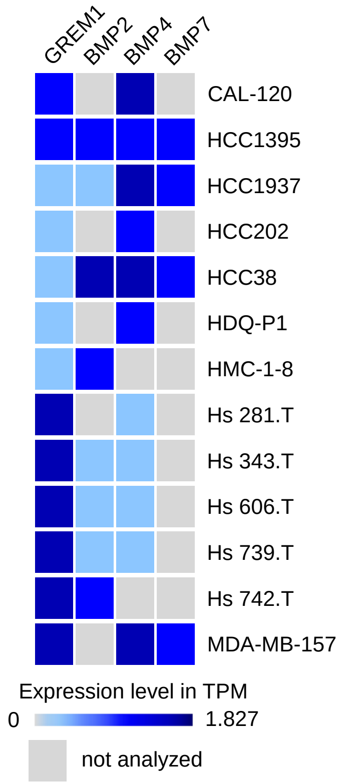

Figure S4. GREM1 is co-expressed with BMPs in several human breast cancer cell lines. Co-expression analysis of GREM1 and selected BMPs (BMP2, BMP4, and BMP7) in human breast cancer cell lines using Expression atlas.
